# Genome‐Driven Analysis Reveals the Biotechnological Potential of a Novel *Paenibacillus* sp. Isolated From Crude Oil

**DOI:** 10.1002/mbo3.70159

**Published:** 2025-11-24

**Authors:** João Victor dos Anjos Almeida, Carlos Miguel Nóbrega Mendonça, Leandro Marcio Moreira, Ricardo Pinheiro Pinheiro de Souza Oliveira, Alessandro de Mello Varani, Mauro de Medeiros Oliveira

**Affiliations:** ^1^ Department of Agricultural and Environmental Biotechnology, College of Agricultural and Veterinary Sciences Sao Paulo State University (UNESP) Jaboticabal Sao Paulo Brazil; ^2^ Department of Biochemical‐Pharmaceutical Technology, Faculty of Pharmaceutical Sciences University of São Paulo São Paulo Sao Paulo Brazil; ^3^ Department of Biological Science, Institute of Exact and Biological Science Federal University of Ouro Preto Ouro Preto Minas Gerais Brazil

**Keywords:** bacteriocins, bioenergy, environmental resilience, functional genomics, industrial microbiology

## Abstract

Microbial biotechnology plays a critical role in addressing environmental challenges and promoting sustainability. Here, we report the complete genome sequencing of *Paenibacillus* sp. strain 210, previously isolated from Brazilian crude oil and known for its levan metabolism and biosurfactant production. With the sequenced genome, we employed bioinformatics tools for assembly and annotation, followed by comprehensive in silico analyses, including phylogenomics, biosynthetic gene cluster (BGC) identification, carbohydrate‐active enzyme (CAZyme) profiling, and metabolic pathway reconstruction. The assembled 5.7 Mb genome harbors four prophage regions and 13 antimicrobial BGCs, including those encoding fusaricidin, paenicidin A, paenilan, paeninodin, and tridecaptin. Phylogenomic analysis combined with average nucleotide identity measurements indicates that this strain does not cluster with any recognized *Paenibacillus* species, supporting its designation as a potential new species. Notably, the identification of 259 CAZyme genes points to a strong capacity for degrading complex polysaccharides (e.g., cellulose, xylan, and pectin), positioning this bacterium as a promising candidate for biofuel production. Furthermore, the presence of complete metabolic pathways for several B vitamins highlights predicted metabolic autonomy supporting microbial interactions, reinforcing their usefulness in soil bioremediation by enhancing nutrient availability. In contrast, incomplete pathways for vitamins B2 and K2 indicate metabolic dependencies that may facilitate syntrophic interactions with other microorganisms. In silico structural analyses of selected hydrolytic enzymes (GH1, GH5, and GH11) reveal homology to functionally validated and crystallized proteins. Collectively, these findings highlight the genetic versatility of *Paenibacillus* sp. strain 210 and its potential for ecosystem restoration, biofuel production, plant growth promotion, and biocontrol.

## Graphical abstract

The novel *Paenibacillus* sp. strain 210, isolated from Brazilian crude oil, its genome encoding 259 carbohydrate‐active enzymes for degrading cellulose, xylan, and pectin, positioning it as a candidate for biofuel production. The strain harbors complete pathways for B‐vitamin biosynthesis and antimicrobial biosynthetic clusters (e.g., fusaricidin and paenilan), alongside plant growth–promoting traits like indole‐3‐acetic acid production, phosphate solubilization, and nitrogen fixation. Phylogenomic and structural analyses confirm its genomic versatility for bioremediation, agriculture, and biocontrol applications.

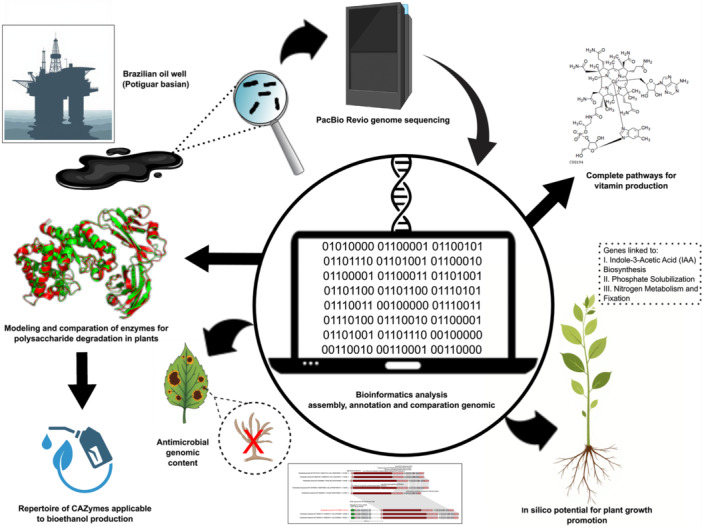

## 1 Introduction

Microbial biotechnology plays a crucial role in addressing urgent global challenges, such as environmental degradation, food security, and public health. These areas form essential pillars in sustainability agendas, aligning with the objectives established in the United Nations' 2030 Agenda. Microorganisms, renowned for their remarkable metabolic diversity and resilience in extreme environments, are thus integral to developing sustainable solutions.

Within this context, certain microbial species play key roles in decomposing complex organic materials. By recycling biological resources, they propel nutrient cycles and help stabilize microbial communities. Polysaccharides, structurally intricate carbon sources, are central to recycling processes across diverse ecosystems—ranging from the human gut to marine environments (Sichert and Cordero 2021). Their degradation yields products that nourish other microorganisms, consequently boosting microbial diversity and functionality (Sichert and Cordero 2021).

Among these polysaccharides, those derived from plants—such as pectin, hemicellulose, and cellulose—constitute critical components of plant cell walls and are abundantly present in agricultural and forestry residues. When efficiently broken down, these polysaccharides produce fermentable sugars vital for bioethanol production (A. P. De Souza et al. 2013). While their complete hydrolysis poses technical challenges, it also offers notable opportunities for advancing sustainable biofuel technologies, waste valorization, and reducing reliance on petroleum‐based energy sources (A. P. De Souza et al. 2013; Zabed et al. 2017).

Enzymatic hydrolysis, employing specialized enzymes (e.g., cellulases and hemicellulases, such as xylanases) to cleave glycosidic bonds, followed by microbial fermentation (Zabed et al. 2017), can transform these biomasses into renewable energy. For example, nonstructural carbohydrates in sugarcane straw degrade gradually, whereas structural carbohydrates often persist (Pagliuso et al. 2021), complicating their utilization. Metabolizing these remaining structural carbohydrates is therefore key to second‐generation (2G) ethanol production (A. P. De Souza et al. 2014), a pursuit that gains urgency given projections that energy crops could meet one‐third of global energy needs by 2050 (Guo et al. 2015).

Moreover, certain microbial species can produce and release essential vitamins in polluted environments, thereby stabilizing microbial communities and supporting the remediation of areas contaminated by oil spills and related pollutants (Babalola 2010; Jonsson and Östberg 2011; Uebanso et al. 2020; Radice et al. 2023). In aquatic settings, these vitamins promote the growth of microalgae used in bioremediation efforts (Radice et al. 2023) or stimulate other microorganisms capable of degrading oil residues in soil (Jonsson and Östberg 2011), aiding ecosystem recovery. Their benefits extend beyond microbial communities to plants (Babalola 2010) and animals (Uebanso et al. 2020), yielding cascading positive effects throughout the ecosystem.

Consequently, microorganisms present innovative solutions to renewable energy challenges, notably by degrading polysaccharides and generating bio‐based compounds suitable as sustainable energy sources. Within this context, a strain of *Paenibacillus* isolated from Brazilian crude oil samples demonstrated in vitro potential for levan metabolism, as well as the production of biosurfactants and bioemulsifiers (Mendonça et al. 2021). These traits suggest promising applications in enhanced oil recovery, accelerated bioremediation via improved hydrocarbon degradation, and the development of biofuels through the conversion of plant polysaccharides into fermentable sugars (Jonsson and Östberg 2011; A. P. De Souza et al. 2014; Mendonça et al. 2021).

*Paenibacillus* species occupy diverse habitats—from soils and plant rhizospheres to extreme environments, such as polar regions and deserts—and exhibit multifunctional traits relevant to agriculture, medicine, and biotechnology (Grady et al. 2016). Originally classified within *Bacillus* based on morphology and endospore formation, *Paenibacillus* with genome sizes ranging from 3.02 to 8.82 Mbp, gene counts from 3064 to 8478, and G + C content between 39% and 59% (Grady et al. 2016).

Species within the genus *Paenibacillus* have emerged as versatile biocontrol agents, offering sustainable solutions against antimicrobial resistance (AMR) and enhancing agricultural productivity. They produce antifungal lipopeptides (e.g., fusaricidin and paenimyxin), hydrolytic enzymes (e.g., chitinases and glucanases), and volatile organic compounds that suppress plant pathogens such as *Fusarium oxysporum*, *Botrytis cinerea*, and *Rhizoctonia solani* (Jeong et al. 2019; Yuan et al. 2022; Dobrzyński and Naziębło 2024). Commercial formulations exploit these traits; for example, products derived from *Paenibacillus polymyxa* control fungal diseases in peppers and strawberries via fusaricidins and polymyxins (S. H. Lee et al. 2013; Jeong et al. 2019; Tsai et al. 2022), while *Paenibacillus elgii* JCK1400, producing pelgipeptins, targets tomato gray mold and wheat rust (Kim et al. 2020). *Paenibacillus* spp. also promote plant growth through indole‐3‐acetic acid (IAA) production, phosphate solubilization, and nitrogen fixation, enhancing soil fertility and crop resilience (Jeong et al. 2019; Do Carmo Dias et al. 2021; Yuan et al. 2022). For instance, *Paenibacillus peoriae* ZBSF16 harbors *ipdC* and *nif* clusters for IAA biosynthesis and nitrogen fixation, while producing fusaricidins, exemplifying its dual agroecological potential (Yuan et al. 2022). Beyond agriculture, species such as *P. peoriae* and *Paenibacillus validus* inhibit clinically relevant pathogens, including *Listeria monocytogenes*, *Candida* spp., and *Staphylococcus aureus*, highlighting their broader biotechnological relevance (Lorentz et al. 2006).

Building upon previous characterization of *Paenibacillus* sp. strain 210 (Mendonça et al. 2021), we used comparative genomics to explore the genetic traits underlying its potential for various biotechnological applications. In this study, we present the complete genome sequence of strain 210 and analyze its genetic determinants of metabolic versatility. Our findings reveal a robust capacity for degrading complex polysaccharides—namely, cellulose, pectin, and xylan—along with complete biosynthetic pathways for B vitamins and genes linked to plant growth promotion and pathogen biocontrol. Collectively, these results underscore the strain's potential for diverse biotechnological applications, most notably in bioethanol production, bioremediation, and plant growth promotion.

## 2 Materials and Methods

### 2.1 Genomic Sequencing of the Isolate

*Paenibacillus* sp. strain 210, isolated from heavy crude oil samples, was collected from a Brazilian oil well in the Potiguar Basin—for further details, see Mendonça et al. (2021)—and stored in the Microbial Biomolecules Laboratory (LBM) collection at the University of São Paulo.

For cultivation, *Paenibacillus* sp. strain 210 was grown on de Man, Rogosa e Sharpe (MRS) agar under microaerophilic conditions at 37°C for 12–14 h, and single colonies were subsequently propagated in liquid MRS medium under the same conditions. The harvested cell pellets were preserved in 20% (v/v) glycerol at −20°C. Genomic DNA was then extracted using the PureLink Genomic DNA Mini Kit (Invitrogen, Thermo Fisher Scientific) according to the manufacturer's instructions, with DNA quality and concentration assessed via spectrophotometry and fluorometry. Sequencing libraries were prepared following the PacBio HiFi workflow, and the genome was sequenced at the Arizona Genomics Institute using the PacBio HiFi REVIO platform.

### 2.2 Genome Assembly and Annotation

The generated reads were assembled with hifiasm v0.19.8 (Feng et al. 2022). Following assembly, the genome was oriented using DNAapler (Bouras et al. 2024), beginning at the *dnaA* gene. Genome quality was assessed with CheckM v1.2.1 (Parks et al. 2015). Putative plasmid sequences were subsequently evaluated using Deeplasmid (Andreopoulos et al. 2022) and PLASMe (Tang et al. 2023), with results cross‐validated against publicly available reference genomes.

Genome annotation was conducted using the National Center for Biotechnology information (NCBI) Prokaryotic Genome Annotation Pipeline (PGAP) v2024‐04‐27 (Tatusova et al. 2016). To refine these annotations, we employed eggNOG‐Mapper v2.1.1 (Cantalapiedra et al. 2021) to map PGAP‐annotated genes to known gene families and InterProScan v5.67‐99.031 (Jones et al. 2014) to predict protein domains, comparing the results with reference genes from corresponding metabolic pathways. Prophage regions were identified using PHASTEST (Wishart et al. 2023), and circular genome representations were generated with the GenoVi pipeline v0.4.3 (Cumsille et al. 2023).

To detect antibiotic resistance genes (ARGs), protein‐coding nucleotide sequences were compared against databases, including NCBI AMRFinderPlus (Feldgarden et al. 2021), CARD (Alcock et al. 2023), ResFinder (Bortolaia et al. 2020), ARG‐ANNOT (Gupta et al. 2014), MEGARES (Doster et al. 2020), and PlasmidFinder (Carattoli et al. 2014) using anti‐biotic resistance screening tool in contigs for antimicrobial resistance or virulence genes (ABRICATE) v1.0.1 (https://github.com/tseemann/abricate). Biosynthetic gene clusters (BGCs) associated with antimicrobial activity were identified using BAGEL v4.0 (van Heel et al. 2018) and antiSMASH v7.1 (Blin et al. 2023), enabling the detection of additional regions linked to other secondary metabolites.

The genomic neighborhoods of the biosynthetic clusters were manually inspected and visualized using LoVis4u v0.1.4.1 (Egorov and Atkinson 2025).

Annotated protein sequences were analyzed for carbohydrate‐active enzyme (CAZyme)‐related functions by mapping them to the CAZy database (Drula et al. 2022) using dbCAN3 (Jinfang et al. 2023), integrating results from HMMER (Potter et al. 2018), dbCAN‐sub, and DIAMOND (Buchfink et al. 2021) to maximize detection accuracy.

### 2.3 In Silico Taxonomic Assignment Placement and Phylogenomic Analysis

The taxonomic classification was determined using genome taxonomy database (GTDB)‐Tk v2 (Chaumeil et al. 2022) in conjunction with data from the Genome Taxonomy Database (GTDB, release 214).

To determine the species affiliation of strain 210, we performed a phylogenomic analysis using 253 reference genomes of *Paenibacillus* species obtained from NCBI data sets. A phylogenomic tree was constructed with IQ‐TREE2 v2.0.7 (Minh et al. 2020) based on a matrix generated by the benchmarking universal single‐copy orthologs (BUSCO) phylogenomics pipeline (https://jamiemcgowan.ie/), using 37 single‐copy core genes (shared by 99.5 of the genomes) identified with BUSCO v5.8.2 (Simão et al. 2015) with the bacillales_odb12 database. As the outgroup, we used *Bacillus cereus* (GCF_002220285.1), *Bacillus subtilis* (GCF_000009045.1), and *Bacillus velezensis* (GCF_000015785.2). The best clustering model, identified by ModelFinder (Kalyaanamoorthy et al. 2017) based on bayesian information criterion, was SYM + I + G4. Branch support was assessed with 1000 bootstrap replicates.

Similarly, we reconstructed the phylogenomic relationships within the clade containing *Paenibacillus* sp. using additional genomes, supporting its evolutionary placement (308 single‐copy core genes shared by all genomes). For this analysis, the best‐fit model was GTR + F + I + *G*4.

For taxonomic support, average nucleotide identity (ANI) values were compared among the closest relatives of *Paenibacillus* sp. We used OrthoANI (I. Lee et al. 2016) for comparisons involving fewer than 10 genomes and ANIclustermap v1.4.0 (https://github.com/moshi4/ANIclustermap) for comparisons involving more than 10 genomes.

### 2.4 Metabolic Pathways Reconstruction

For the reconstruction of degradation and biosynthesis pathways, KEGG Orthology (KO) terms annotated by eggNOG‐Mapper were referenced against the Kyoto Encyclopedia of Genes and Genomes (KEGG) database and pathways (Kanehisa and Goto 2023).

The metabolic pathways consulted were arabinose (Watanabe et al. 2006; W. R. De Souza 2013), cellulose (KEGG: map00500), fructans (KEGG: ec00051; Buntin et al. 2017), pectin (KEGG: map00040), raffinose (KEGG: map00052), and xylan (KEGG: rn00040 and RAST—Overbeek et al. 2014) for plant polysaccharides, and isopentenyl pyrophosphate (IPP) (Jang et al. 2011), B1/B5/B12/K2 (KEGG: map01240), B2 (KEGG: map00740), B3 (Luo et al. 2023), precursor of B6 (KEGG: rn00750), B7 (KEGG: rn00780), and B9 (KEGG: map00790), for vitamins and precursors.

### 2.5 Molecular Modeling of Candidate Enzymes

Signal peptides were first identified and removed, if present, using SignalP 6.0 (Teufel et al. 2022) before molecular modeling. The refined sequences were then used for structure prediction with the AlphaFold v3 platform (Abramson et al. 2024), ensuring accurate functional validation of the selected enzymes. We used the SAVES 6.1 platform (https://saves.mbi.ucla.edu/) for structural evaluations, and the models were visualized using PyMOL v3.1.3.1. Additionally, predictions were cross‐referenced with UniProt (https://www.uniprot.org/) to ensure consistency with experimental data. Three‐dimensional structure superposition was calculated using the FATCAT 2.0 tool (Z. Li et al. 2020). All figures were edited with Inkscape v1.4 (https://inkscape.org).

Unless otherwise specified, all software tools utilized were run using their default parameters.

## 3 Results

### 3.1 Genomic Features and Species‐Level Definition of the *Paenibacillus* sp. Strain 210

**Figure 1:**
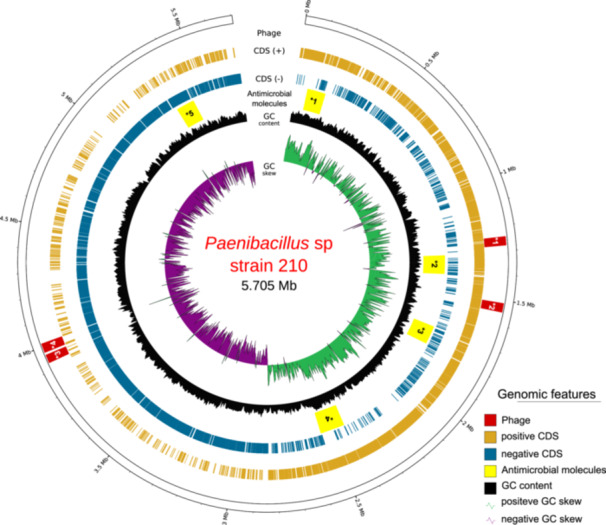
Circular representation of the *Paenibacillus* sp. strain 210 genome (5.705 Mb), where the rings display different genomic features. The outermost ring indicates the identified prophage regions (in the dark red with *1 questionable completeness with NC_03094, *2 intact completeness with NC_028805, *3 questionable completeness with NC_048651, and *4 questionable completeness with NC_048762), followed by the distribution of coding sequences (CDS) on the positive strand (in golden beige) and the negative strand (in blue). The next ring highlights gene clusters associated with the production of antimicrobial molecules (in yellow with *1 fusaricidin B, *2 paeninodin, *3 paenilan, 4* tridecaptin, and *5 paenicidin A). Further inward, the GC content graph (in black) reflects variations in nucleotide composition throughout the genome. At the center, the positive GC skew (in green) and negative GC skew (in purple) are shown.

A total of 928,813 HiFi reads were generated and assembled into a single contig representing a complete circular genome spanning 5.705 Mb, with no detectable plasmids (accession number CP160863). The genome has a guanine and cytosine (GC) content of 46.5% and an average sequencing coverage of 86× (Figure 1).

The genome harbors four prophage regions and five regions associated with antimicrobial molecules production (Figure 1). Annotation with the NCBI PGAP identified a total of 5178 genes (Table 1). Quality assessment by CheckM indicated 99.72% completeness, while BUSCO analysis confirmed 99.30% completeness.

**Table 1:** Genomic features of *Paenibacillus* sp. strain 210.

Features	*Paenibacillus* sp. 210
Genomic size (bp)	5,705,131
Sequencing depth (mean)	86×
GC content (%)	46.5
Genes (total)	5170
CDSs (total)	5040
tRNA	92
5S rRNA	11
16S rRNA	11
23S rRNA	11
ncRNA	4
Pseudogenes	123
CRISPR arrays	9
Antibiotic resistance‐related genes (ABRICATE)	‐ 1× (AGly)aadE‐Pp (aminoglycoside)
	‐ 1× (Rif)rphD (rifamycin)
Virulence‐related genes	‐ Hemolysin III family protein^a^ ‐ 3x Hemolysin family protein^a^
CheckM (completeness/contamination)	99.72/1.25
BUSCOs (completeness)	99.3

Abbreviations: CDS, distribution of coding sequence; CRISPR, clustered regularly interspaced short palindromic repeats; ncRNA, noncoding RNA; tRNA, transfer RNA.

a The annotated genes were identified in the Prokaryotic Genome Annotation Pipeline but were not detected using ABRICATE and its accessory databases.

Taxonomic classification using GTDB‐Tk was not achieved, as the ANI values failed to meet the program's threshold. The closest relatives identified included *Paenibacillus kribbensis* (GCF 002240415.1; 92.91% ANI), *P. peoriae* (GCF 000236805.1; 92.57% ANI), *Paenibacillus brasilensis* (GCF 009363115.1; 91.1% ANI), and *P. polymyxa* (GCF 001719045.1; 87.35% ANI), among others.

This ambiguity was corroborated by complementary BLAST analysis in the NCBI repository using the *rpoB* (Da Mota et al. 2004) gene sequence—considering the inconclusive classification by the 16S sequence evaluated in a previous study, accession number MW577094 (Mendonça et al. 2021). For that gene, the top hits were *P. kribbensis* PS04 (GCF 013394225.1; 99% coverage and 96.56% identity), *P. brasilensis* KACC 13842 (99% coverage and 96.50% identity), *P. peoriae* KCTC 3673 (GCF 009363115.1; 99% coverage and 96.36% identity), and *P. polymyxa* HY96‐2 (GCF 002893885.1; 100% coverage and 94.42% identity).

**Figure 2:**
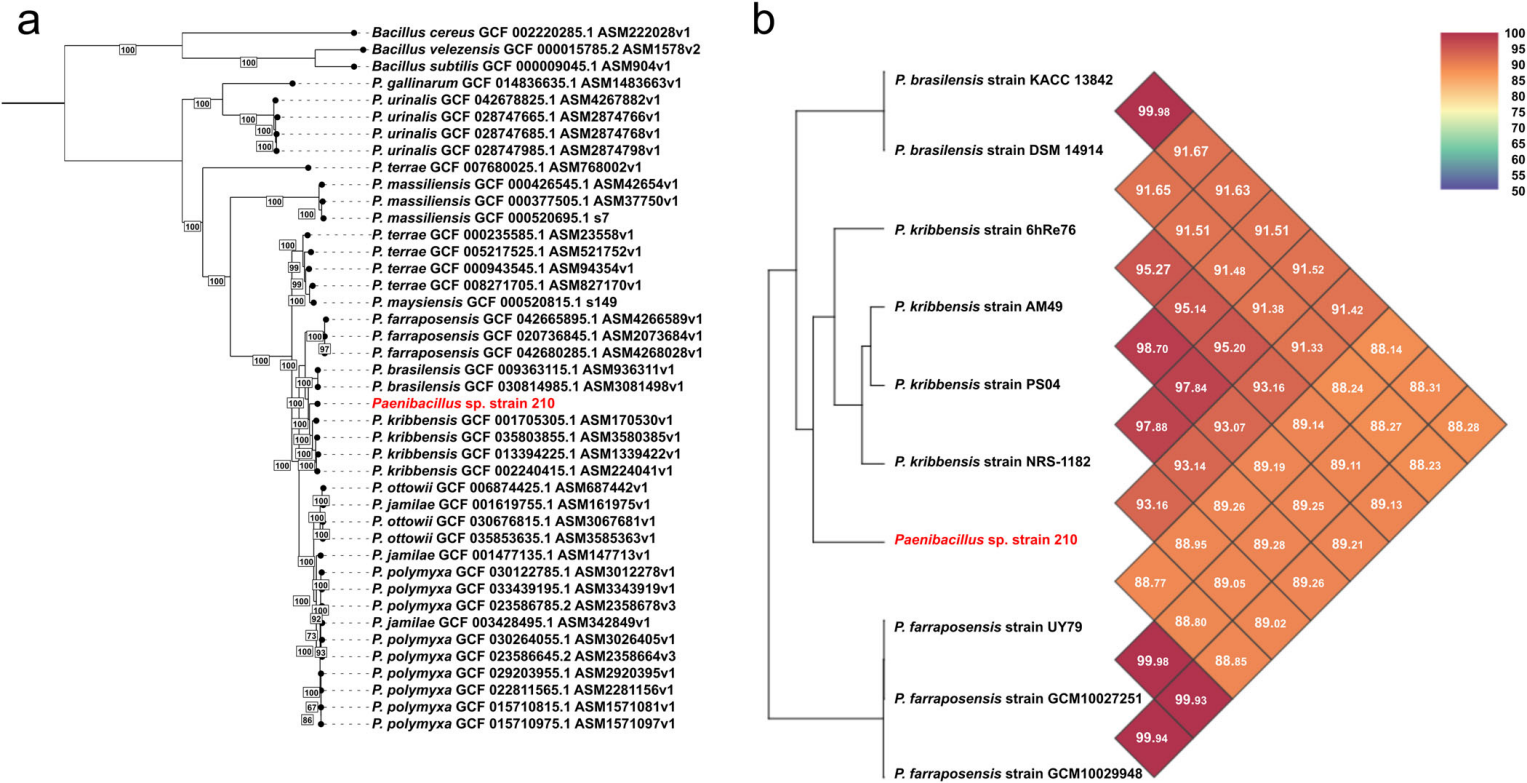
(a) Phylogenetic tree showing the relationships between strain 210 (red) and *Paenibacillus* species. (b) Heatmap generated by OrthoANI, representing the average nucleotide identity (ANI) values between the genomes of the clade highlighted in green in the phylogeny, with the color gradient indicating the degree of similarity.

We then reconstructed the phylogenomic relationships between our strain and reference genomes of *Paenibacillus* species (Figures 2a and Supporting Information Figures A1 and A2 and Table A1), which enabled a more precise identification of its position within the *Paenibacillus* genus. Together with the ANI cluster data, our analyses indicate that the distance on average genome identities to strain 210 is less than 93% identity, allowing us to classify it as a new species (Figure 2b and Supporting Information Figure A3).

Comparing the genomes used in the ANI analysis of the *Paenibacillus* sp. strain 210 clade, we identified reference species associated with both soil and plants, including endophytic (e.g., *Paenibacillus farraposensis*) and rhizospheric (e.g., *Paenibacillus maysiensis*) forms (Table 2). Furthermore, most genomes lacked identifiable plasmids and exhibited distinct patterns in bacteriophage region predictions. The distribution of AMR and phage regions for the other reference genomes can be found in Supporting Information Tables A2 and A3, respectively.

**Table 2:** Comparison of the nine species phylogenomically closest of *Paenibacillus* sp. 210, detailing the source and geographic location of isolation, as well as the detection of prophage regions, the presence of plasmids, and resistance genes.

Features	Psp	Pbr	Pkr	Ppe	Pfa	Pot	Ppo	Pja	Pte	Pma
Country	Brazil	South Korea	South Korea	South Korea	Uruguay	EUA	China	India	South Korea	China
Isolation source	Crude oil	Soil	Soil	Soil	Nodule endophyte	anaerobic digestate	Soil	Rice seed	Soil	Corn rhizosphere
Plasmid	0	1	0	0	0	0	1	0	0	0
AMR										
(Agly)aadE‐Pp	1	1	0	0	0	1	0	1	0	0
clbB	0	1	0	0	0	0	0	0	0	0
RPH	1	1	1	1	0	1	1	1	1	1
Prophage										
*Aeribacillus* phage AP45 (NC_048651)	quest	0	inc	0	0	quest + int	int	0	0	
*Bacillus* phage BalMu‐1 copy 1 (NC_030945)	quest	quest	int	quest	quest	quest	quest	0	quest	quest
*Brevibacillus* phage Jenst (NC_028805)	int	0	int	int	int	0	0	0	int	int
*Bacillus* phage vB_BtS_B83 (NC_048762)	quest	0	0	quest	0	0	0	0	0	int
*Brevibacillus* phage Jimmer2 (NC_041976)	0	int	0	0	int	0	0	0	0	0
Deep‐sea thermophilic phage D6E (NC_019544)	0	quest	0	0	0	0	0	0	quest	0
*Paenibacillus* phage Xenia (NC_028837)	0	int	0	0	inc	0	inc	0	0	0
*Lactobacillus johnsonii* prophage Lj771 (NC_010179)	0	inc	0	0	0	0	0	0	0	0
*Clostridium* phage phiCT453A (NC_028991)	0	quest	0	0	0	0	0	0	0	0
Bacteriophage lily (NC_028841)	0	0	0	0	int	0	0	0	0	0
*Geobacillus* phage TP‐84 (NC_041918)	0	0	0	0	inc	0	0	0	0	0
*Paenibacillus* phage Harrison (NC_028746)	0	0	0	0	inc	0	0	0	0	0
*Paenibacillus* phage PG1 (NC_021558)	0	0	0	0	inc	0	0	0	0	0
*Bacillus* phage Fah (NC_007814)	0	0	0	0	inc	0	0	0	0	0
*Bacillus* phage BM5 (NC_029069)	0	0	0	0	0	2x_quest	0	0	0	inc
*Brevibacillus* phage Sundance (NC_028749)	0	0	0	0	0	0	int	0	0	0
*Geobacillus* phage GBSV1 (NC_008376)	0	0	0	0	0	0	int	0	0	0
*Paenibacillus* phage Tripp (NC_028930)	0	0	0	0	0	0	0	quest	quest	0
*Bacillus* phage Mgbh1 (NC_041879)	0	0	0	0	0	0	0	quest	0	0
*Bacillus* phage SP10 (NC_019487)	0	0	0	0	0	0	0	0	0	int
*Paenibacillus* phage HB10c2 (NC_028758)	0	0	0	0	0	0	0	0	0	inc

Abbreviations: AMR, antimicrobial resistance; inc, incomplete; int, intact; Pbr, *Paenibacillus brasilensis*; Pfa, *Paenibacillus farraposensis*; Pja, *Paenibacillus jamilae*; Pkr, *Paenibacillus kribbensis*; Pma, *Paenibacillus maysiensis*; Pot, *Paenibacillus ottowii*; Ppe, *Paenibacillus peoriae*; Ppo, *Paenibacillus polymyxa*; Psp, *Paenibacillus* sp. strain 210; Pte, *Paenibacillus terrae*; quest, questionable; RPH, RNase tRNA nucleotidyltransferase.

Regarding the shared biosynthetic regions for antimicrobial molecules in *Paenibacillus* sp., fusaricidin B (Supporting Information Figure A4), paeninodin (Supporting Information Figure A5), and tridecaptin (Supporting Information Figure A6) are conserved across the nine taxonomically related species (Supporting Information Table A4). However, paenilan (Supporting Information Figure A7) is shared with more distant species, according to the phylogenomic relationships we reconstructed, but is absent in the closest genomes. No predictions for paenicidin were found in the closest species clade, except in *Paenibacillus* sp. (although its characterization has already been observed in *P. polymyxa* NRRL B‐30509 in literature, Lohans et al. 2012). Additionally, *Paenibacillus tianmuensis* (GCF_900100345.1) showed predictions for paenicidin but with an incomplete biosynthetic cluster (Supporting Information Figure A8).

### 3.2 CAZyme Profiles of *Paenibacillus* sp. and Other *Paenibacillus* sp. Genomes

**Figure 3:**
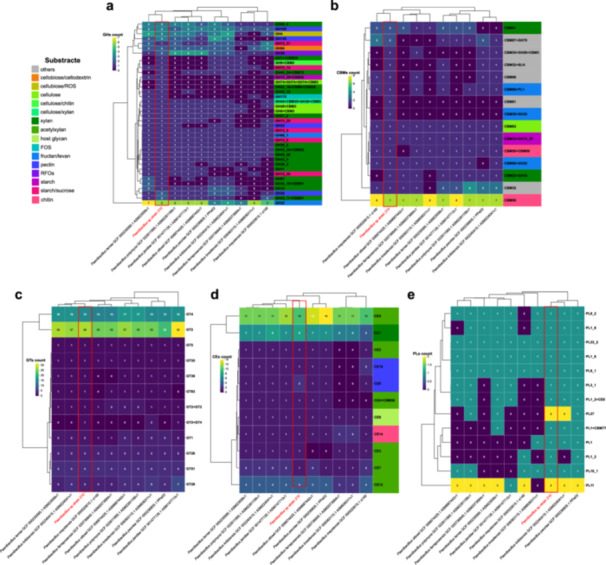
CAZyme profiles associated with polysaccharide degradation in different *Paenibacillus* genomes, specifically within the phylogenomic clade most closely related to *Paenibacillus* sp. (highlighted in red). Heatmaps are provided for: GH (a), CBM (b), GT (c), CE (d), and PL (e). The dendrogram reflects the similarity between CAZyme profiles based on the presence and abundance, calculated using Euclidean distance (top, species relationships; left, CAZyme family relationships). AAs, auxiliary activities; CAZyme, carbohydrate‐active enzyme; CBMs, carbohydrate‐binding modules; CEs, carbohydrate esterases; FOS, fructooligosaccharides; GHs, glycoside hydrolases; GT, glycosyltransferase; PLs, polysaccharide lyases; RFO, raffinose family oligosaccharides.

Using dbCAN3, we identified a diverse CAZyme repertoire in *Paenibacillus* sp. strain 210, including glycoside hydrolases (GHs), carbohydrate esterases (CEs), polysaccharide lyases (PLs), carbohydrate‐binding modules (CBMs), and auxiliary activities (AAs). Strain 210 encodes 259 CAZymes (Figure 3), a genomic feature consistent with the genus *Paenibacillus*, as shown in comparative analyses with reference genomes (Supporting Information Table A5). Substrate specificity predictions linked these enzymes to the degradation of cellulose, xylan, and pectin, corroborated by the abundance of families associated with these polysaccharides in the heatmap (Figure 3).

The GH profile of strain 210 (Figure 3a) revealed GH43 as the most prominent family, with 12 predicted alpha‐arabinofuranosidases and beta‐xylosidases. These enzymes, critical for xylan degradation, were frequently coupled with CBM36 and CBM91 (indicated by adjacent color blocks in Figure 3a). GH32 (green, abundance level 4), associated with fructan metabolism (e.g., inulinases), was also highly represented, with eight entries linked to CBM66 and CBM38 (Figure 3b). Among the compared genomes, strain 210 exhibited the highest GH diversity (55 families), followed by *Paenibacillus terrae* (52 families), as visualized by the extended teal and green blocks in the heatmap (Figure 3a). In the CEs group, strain 210 did not dominate in CE abundance (Figure 3b), its profile highlighted enzymes targeting acetylxylan (orange gradients, linked to CE1 and CE4), associated with xylanolytic activity. For glycosyltransferases (GTs), strain 210 presented 61 GT families (Figure 3c), comparable to most genomes of the clade. *Paenibacillus jamilae* presented the highest number of GTs (69 families). Finally, for the PLs group, strain 210 exclusively encoded PL9 and PL10 (purple blocks, Figure 3e), associated with pectate/pectin degradation, highlighting its potential for plant biomass breakdown.

### 3.3 Characterization of Identified Metabolic Pathways

A total of 16 metabolic pathways were investigated by reconstructing these processes through KO annotations using eggNOG‐mapper. To validate our findings, we compared shared protein domains between reference sequences cataloged in the KEGG database for each pathway. This approach enabled the identification of sequences with common domains, thereby reinforcing the accuracy of the pathway assignments (Supporting Information Table A6).

**Figure 4:**
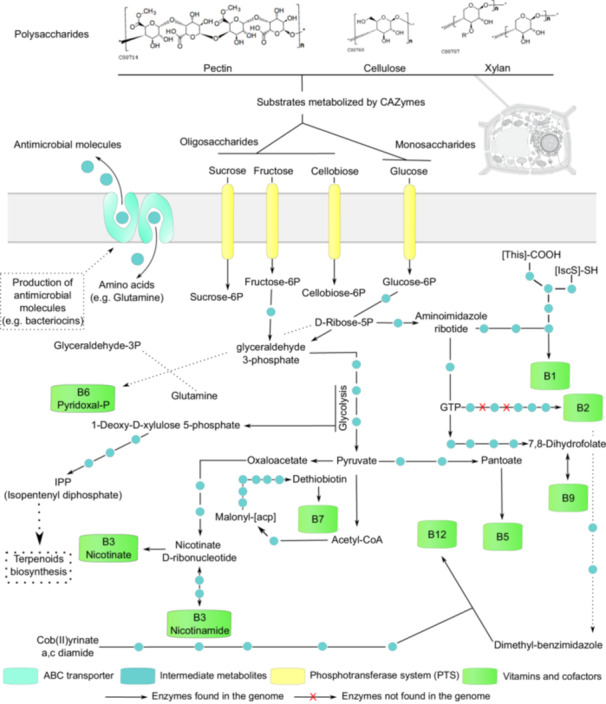
Simplified scheme of the metabolic pathways verified in *Paenibacillus* sp. strain 210. Representations used are available at polysaccharides (https://www.genome.jp/kegg/compound/) and plant cell structure (https://www.swissbiopics.org/). ABC, ATP‐binding cassette; ATP, adenosine triphosphate; CAZyme, carbohydrate‐active enzyme; GTP, guanosine triphosphate; IPP, isopentenyl pyrophosphate.

Through in silico reconstruction of metabolic pathways in our *Paenibacillus* sp. strain 210, we identified genomic features suggesting a strong capacity to degrade various polysaccharides, especially plant‐derived ones such as fructans, as well as structural carbohydrates like xylan and pectin (Supporting Information Table A6). We also found evidence of pathways linked to B‐complex vitamin biosynthesis and IPP formation—an important intermediate in terpenoid biosynthesis—highlighting this isolate's potential for diverse biochemical functions (Supporting Information Table A6). In *Paenibacillus* sp., complete pathways were identified for the degradation of polysaccharides, including inulin, levan—linked to the production of this compound observed in the strain (Mendonça et al. 2021)—xylan, cellulose, and pectin (Figure 4). Regarding vitamin biosynthesis, *Paenibacillus* sp. strain 210 possesses complete pathways for B1, B3, B5, B6, B7, B9, and B12, indicating its ability to synthesize these vitamins, as well as IPP (isopentenyl diphosphate), which is associated with terpenoid production. In contrast, the pathways for B2 and K2 are incomplete, suggesting potential limitations in their biosynthesis or dependence on external sources (Figure 4).

### 3.4 In Silico Structural Comparison of Enzymes Involved in Cellulose, Pectin, and Xylan Metabolism Pathways

Functional annotation of complex carbohydrate metabolism in *Paenibacillus* sp. strain 210, based on EC numbers and KO terms, enabled the in silico reconstruction of cellulose, pectin, and xylan degradation pathways. Enzyme structures predicted by AlphaFold 3 were compared with experimentally solved references using FATCAT, providing mechanistic insight into the strain's polysaccharide‐degrading capacity.

**Figure 5:**
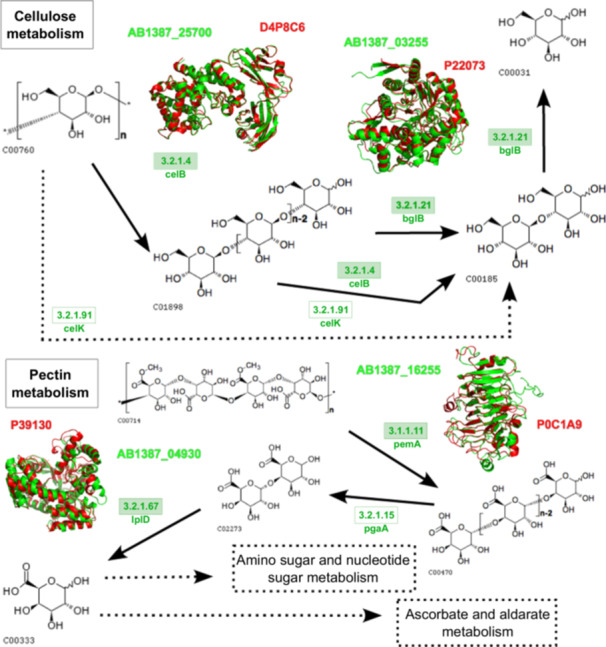
Molecular alignment of protein relationships with cellulose and pectin degradation pathways from *Paenibacillus* sp. (in green) with the references structures in the PDB (in red), reference pathway for cellulose metabolism in KEGG: map00500, and pectin in KEGG: map00040. KEGG, Kyoto Encyclopedia of Genes and Genomes.

Key enzymes analyzed included endoglucanase B, beta‐glucosidase A, pectinesterase A, alpha‐galacturonidase, endo‐1,4‐beta‐xylanase A, beta‐xylosidase, and xylulose kinase. Endoglucanase B (GH5, E.C. 3.2.1.4; ABI1387_25700) showed high structural conservation with a *Bacillus* sp. cellulase protein data bank (PDB): 5E09; with 531 aligned positions and a root‐mean‐square deviation (RMSD) of 1.06 Å, supported by 90.8% of residues in favored Ramachandran regions (Figure 5 and Supporting Information Figure A9 and Table A7). Similarly, beta‐glucosidase (GH1; ABI1387_03255) aligned with *P. polymyxa* (1BGG), showing 448 equivalent positions and RMSD of 1.91 Å (Supporting Information Table A7).

**Figure 6:**
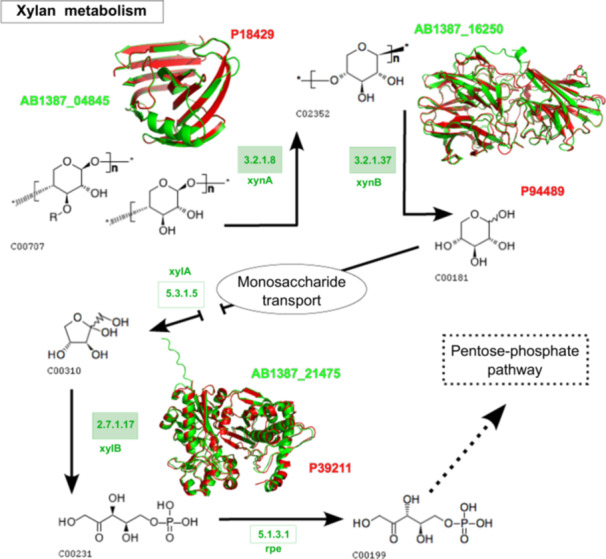
Molecular alignment of protein relationships with xylan degradation pathways from *Paenibacillus* sp. (in green) with the references structures in the PDB (in red). reference pathway for xylan metabolims in KEGG: rn00040 and RAST: https://rast.nmpdr.org/seedviewer.cgi?page=Subsystems&subsystem=Xylose_utilization. KEGG, Kyoto Encyclopedia of Genes and Genomes; RAST, Rapid Annotation of Subsystems Technology.

Comparable structural conservation was observed for pectin metabolism enzymes, *pemA* (AB1387_16255 vs. 2NSP, *Dickeya dadantii*) and lplD (AB1387_04930 vs. 3FEF, *B. subtilis*), as well as endo‐1,4‐beta‐xylanase A (GH11, E.C. 3.2.1.8; AB1387_04845 vs. 1YAW, *B. subtilis*), reveal high three‐dimensional similarity (Figures 5 and 6). Ramachandran analysis shows 89.4% of residues in favored regions and 10.6% in allowed regions, confirming reliable structural models (Supporting Information Figure A9 and Table A7). The xylanase structures share 184 equivalent positions with an RMSD of 0.18 Å and no significant distortions (Supporting Information Table A7).

### 3.5 Plant Growth Promotion Mechanisms in *Paenibacillus* sp. Strain 210

The plant growth–promoting (PGP) capabilities of *Paenibacillus* sp. strain 210 are supported by a suite of genes involved in auxin biosynthesis, phosphate solubilization, nitrogen metabolism, and nitrogen fixation. These genetic traits enable the bacterium to enhance plant nutrient acquisition, stress tolerance, and overall growth.

IAA production, a key phytohormone for root development, involves the tryptophan‐dependent pathway with genes such as *aroC*, *aroH*, *aroF*, *trpA–D*, and *ipdC*, enabling chorismate and tryptophan synthesis and facilitating decarboxylation reactions critical for IAA biosynthesis (Table 3).

**Table 3:** Indole‐3‐acetic acid (IAA) biosynthesis genes in *Paenibacillus* sp. strain 210.

Gene	Product	Role in IAA biosynthesis	Gene ID
*aroC*	Chorismate synthase	Converts 5‐enolpyruvylshikimate‐3‐phosphate to chorismate	AB1387_14830
*aroH*	Chorismate mutase	Catalyzes the conversion of chorismate to prephenate	AB1387_14820
*aroF*	3‐Deoxy‐7‐phosphoheptulonate synthase	Key enzyme in the shikimate pathway	AB1387_07710
*trpA*	Tryptophan synthase subunit alpha	Converts indole‐3‐glycerol phosphate to tryptophan	AB1387_14790
*trpB*	Tryptophan synthase subunit beta	Converts indole‐3‐glycerol phosphate to tryptophan	AB1387_14795
*trpS*	Tryptophan‐transfer RNA ligase	Activates tryptophan for protein synthesis	AB1387_20675
*trpC*	Indole‐3‐glycerol phosphate synthase	Catalyzes indole‐3‐glycerol phosphate synthesis	AB1387_14805
*trpD*	Anthranilate phosphoribosyltransferase	Converts anthranilate to phosphoribosylanthranilate	AB1387_14810
*trpE*	Anthranilate synthase component I	Synthesizes anthranilate from chorismate	AB1387_14815
*trpG*	Glutamine amidotransferase	Supports anthranilate synthase activity	AB1387_18125
*trpCF*	Phosphoribosylanthranilate isomerase	Converts phosphoribosylanthranilate to carboxyphenylaminodeoxyribulose phosphate	AB1387_14800
*ipdC*	Thiamine pyrophosphate‐binding protein	Involved in indole‐3‐pyruvate decarboxylation	AB1387_00400

Phosphate solubilization in *Paenibacillus* sp. strain 210 is potentially supported by genes encoding phosphatases (*phoN*, phoA), transporters (*phnC/D/E* and *ptsS/C/A/B*), and the regulator phoU (Table 4).

**Table 4:** Phosphate solubilization genes in *Paenibacillus* sp. strain 210.

Gene	Product	Role in phosphate solubilization	Gene ID
*phoN*	Phosphatase PAP2 family protein	Hydrolyzes organic phosphate esters	AB1387_21265
*iap*	Aminopeptidase	Degrades organic phosphate compounds	AB1387_11915
*phoA*	Alkaline phosphatase	Releases inorganic phosphate from organic sources	AB1387_21945
*phnE*	Phosphonate ABC transporter permease	Transports phosphonates	AB1387_21795
*phnD*	Phosphonate ABC transporter substrate‐binding protein	Binds extracellular phosphonates	AB1387_21780
*phnC*	Phosphonate ABC transporter ATP‐binding protein	Energizes phosphonate transport	AB1387_21785
*ptsS*	Phosphate ABC transporter substrate‐binding protein	Binds extracellular phosphate	AB1387_08280
*ptsC*	Phosphate ABC transporter permease subunit	Transports phosphate across the membrane	AB1387_08285
*ptsA*	Phosphate ABC transporter permease PstA	Transports phosphate across the membrane	AB1387_08290
*ptsB*	Phosphate ABC transporter ATP‐binding protein	Energizes phosphate transport	AB1387_08295
*phoU*	Phosphate signaling complex protein	Regulates phosphate uptake	AB1387_08370

Abbreviations: ABC, ATP‐binding cassette; ATP, adenosine triphosphate; PAP2, type II phosphatidic acid phosphatase.

Nitrogen assimilation in *Paenibacillus* sp. strain 210 is potentially supported by genes involved in nitrate reduction (*narG/H/I/J* and *nirB/D*), nitrate/nitrite transport (*narK* and *nirC*), and ammonium uptake (amtB) (Table 5).

**Table 5:** Nitrogen metabolism (nitrate transport/reduction) genes in *Paenibacillus* sp. strain 210.

Gene	Product	Role in nitrogen metabolism	Gene ID
*narI*	Respiratory nitrate reductase subunit gamma	Reduces nitrate to nitrite	AB1387_08500
*narJ*	Nitrate reductase molybdenum cofactor assembly chaperone	Assists in nitrate reductase assembly	AB1387_08495
*narH*	Nitrate reductase subunit beta	Catalyzes nitrate reduction	AB1387_08490
*narG*	Nitrate reductase subunit alpha	Catalyzes nitrate reduction	AB1387_08485
*narK*	Nitrate/nitrite transporter	Transports nitrate/nitrite across membranes	AB1387_08465
*nirD*	Nitrite reductase small subunit	Reduces nitrite to ammonia	AB1387_03300
*nirC*	Nitrate/nitrite transporter	Transports nitrite	AB1387_16035
*nirB*	Nitrite reductase large subunit	Reduces nitrite to ammonia	AB1387_03295
*amtB*	Ammonium transporter	Imports ammonium into the cell	AB1387_08985

The *nif* gene cluster in *Paenibacillus* sp. strain 210 (Table 6) potentially enables atmospheric nitrogen fixation. Key genes, including *nifH*, *nifD/K*, and *nifB/E/N*, along with *hesA* for cofactor biosynthesis, may allow the reduction of N₂ to NH₃, supplying plants with a direct nitrogen source.

**Table 6:** Nitrogen fixation genes in *Paenibacillus* sp. strain 210.

Gene	Product	Role in nitrogen fixation	Gene ID
*nifH*	Nitrogenase iron protein	Electron donor for nitrogenase	AB1387_05115
*nifN*	Nitrogenase cofactor biosynthesis protein	Assembles FeMo‐cofactor	AB1387_05135
*nifB*	Nitrogenase cofactor biosynthesis protein	Synthesizes FeMo‐cofactor precursor	AB1387_05110
*nifD*	Nitrogenase molybdenum–iron protein alpha chain	Catalyzes N₂ reduction	AB1387_05120
*nifU*	Nitrogenase Fe─S cluster assembly protein	Assists in Fe─S cluster formation	AB1387_20555
*nifE*	Nitrogenase cofactor biosynthesis protein	Assembles FeMo‐cofactor	AB1387_05130
*nifK*	Nitrogenase molybdenum–iron protein subunit beta	Catalyzes N₂ reduction	AB1387_05125
*nifX*	Nitrogen fixation protein	Binds FeMo‐cofactor intermediates	AB1387_05140
*hesA*	HesA/MoeB/ThiF family protein	Involved in molybdopterin biosynthesis	AB1387_05145

The search for this gene set was also extended to species that are phylogenomically related to strain 210 (Supporting Information Table A8).

## 4 Discussion

*Paenibacillus* sp. strain 210, isolated from crude oil–contaminated soil in Brazil, exhibits genomic traits reflecting ecological specialization and biotechnological potential. Its genome reveals taxonomic novelty, enzymatic capabilities for polysaccharide degradation, antimicrobial biosynthesis, and PGP mechanisms, providing a foundation for understanding its adaptation to extreme environments and potential applied uses.

### 4.1 Genomic Basis for Taxonomic Distinction and Ecological Adaptation in *Paenibacillus* sp. Strain 210

*Paenibacillus* sp. strain 210 represents a novel species within the genus, as evidenced by phylogenomic analysis and ANI values below 93% compared with its closest relatives (*P. kribbensis, P. peoriae*, and *P. brasilensis*) (Da Mota et al. 2004; Xu et al. 2014; Do Carmo Dias et al. 2021; Yuan et al. 2022).

Considering that genomic approaches are robust enough for statistically supported taxonomic assignments—exemplified by ANI values below 95% as a species threshold (Chun et al. 2018)—and given the absence of distinctive sequences in universal bacterial markers such as 16S rRNA (Mendonça et al. 2021) or genus‐specific markers (Da Mota et al. 2004), our findings, integrated with phylogenomic analyses based on 308 single‐copy core genes shared with the closest related genomes, provide a plausible inference for the designation of a new taxon, as similarly demonstrated in other studies (Da Silva et al. 2022; Xue et al. 2023; Kong et al. 2025).

The 5.705 Mb circular genome, characterized by a GC content of 46.5% and devoid of plasmids, harbors four intact prophage regions and 13 BGCs linked to antimicrobial production. These features distinguish strain 210 from established *Paenibacillus* species (Supporting Information Tables A1–A4). While paenilan—a lantibiotic with anti‐Gram‐positive activity—is shared with *P. polymyxa* E681 (Jeong et al. 2019), its absence in other clade members underscores strain 210's phylogenetic novelty (S. H. Lee et al. 2013; Jeong et al. 2019; Tsai et al. 2022; Dobrzyński and Naziębło 2024). The strain 210 displayed nitrogen fixation (*nif*), AIA and phosphate solubilization genes on the chromosome in contrast to plasmid‐bearing *P. polymyxa* strains, suggesting niche specialization in hydrocarbon‐rich environments (Jeong et al. 2019; Do Carmo Dias et al. 2021; Yuan et al. 2022). Such genomic traits align with its isolation from crude oil–contaminated soil, positioning it as a candidate for bioremediation strategies aimed at restoring microbial communities in polluted ecosystems (Y. Li, Li, et al. 2022; Dewiyanti et al. 2024; T. Li et al. 2024; Polyak et al. 2024).

### 4.2 CAZymes and Structural Basis of Biomass Deconstruction

*Paenibacillus* sp. strain 210 encodes 259 CAZymes, surpassing related species like *P. terrae* (52 GHs) and *P. polymyxa* (Jeong et al. 2019). Key families include GH5 (cellulases), GH43 (xylanases/arabinofuranosidases), and PL9/PL10 (pectinases), which enable degradation of cellulose, xylan, and pectin—critical for processing plant biomass in degraded soils. GH43 enzymes, coupled with CBMs (CBM36/91), facilitate hemicellulose deconstruction, while PL9/PL10 pectinases may enhance root penetration in compacted soils (He et al. 2009; Paës et al. 2012; Dewiyanti et al. 2024).

The expanded GH diversity observed in strain 210 (55 families) compared with other members of the genus reflects potential ecological advantages, particularly in environments rich in complex polysaccharides derived from plant residues (Table 5). This genomic profile is consistent with the well‐documented ecological versatility of *Paenibacillus* spp. (Grady et al. 2016; Jeong et al. 2019; Do Carmo Dias et al. 2021; Yuan et al. 2022), which frequently inhabit the rhizosphere and contribute to organic matter turnover. The presence of PL9 and PL10 in strain 210 further underscores a distinctive enzymatic trait that may be associated with a specialized ecological niche or enhanced plant–microbe interactions, given their rare occurrence in other members of the genus (Table 5).

Although this study is based on in silico genomic analysis, the CAZyme repertoire of strain 210 provides a strategic foundation for future wet‐lab validation. Targeted enzymatic assays focusing on GH5, GH11, and PL9/10 families could assess substrate specificity, activity across environmental conditions, and synergistic lignocellulose degradation. Heterologous expression and biochemical characterization may further identify enzymes with industrial potential for biofuel production, bioremediation, and agricultural applications.

Structural modeling of the GH5 cellulase (ABI1387_25700) and GH11 xylanase (AB1387_04845) using AlphaFold 3 confirmed conserved catalytic domains (RMSD: 1.06 and 0.18 Å, respectively), validating their functional parity with homologs in *Bacillus* spp. (Paës et al. 2012). These enzymatic capabilities mirror *P. polymyxa* E681's role in root exudate processing (Jeong et al. 2019) but underscore strain 210's superior potential for lignocellulosic biorefinery applications, such as bioethanol production from sugarcane waste (He et al. 2009).

The use of structural modeling and comparison, in combination with primary functional annotation and domain‐based validation, provides a multi‐layered in silico approach to assess enzyme functionality. By integrating these steps, we enhance the reliability of our predictions regarding the potential phenotypic roles of the candidate enzymes, while acknowledging that environmental and regulatory factors also influence gene expression and phenotype, offering a robust framework for interpreting their biological significance within the context of the genome.

### 4.3 Gene‐Encoded Metabolic Versatility Supporting Microbial Interactions and Ecological Resilience

The genome contains complete pathways for B‐complex vitamins (B1, B3, B5, B6, B7, B9, and B12) and IPP, a terpenoid precursor. Vitamin B12 synthesis, rare in *Paenibacillus*, may stabilize microbial consortia in oil‐polluted soils by supporting auxotrophic organisms (Yuan et al. 2022). The strain 210 fructan metabolism (GH32 + CBM66) enables energy harvesting from plant‐derived levan, akin to *P. peoriae* ZBSF16 (Yuan et al. 2022), while incomplete pathways for B2 and K2 suggest metabolic dependencies that foster syntrophic interactions (Yuan et al. 2022).

It is important to note that while the genome encodes these pathways, gene presence does not necessarily imply constitutive expression. Environmental conditions, nutrient availability, interspecies interactions, and regulatory mechanisms can strongly influence whether these genes are expressed and their corresponding enzymes are functional (Bervoets and Charlier 2019; Wang et al. 2023; Sinha et al. 2025). Therefore, the metabolic versatility inferred from genomic data represents potential capabilities that require experimental validation under relevant ecological conditions. These traits, when expressed, could align with bioremediation strategies where vitamin secretion and polysaccharide degradation synergize to rejuvenate microbial communities in contaminated environments (Guo et al. 2015).

### 4.4 Integrative Genomic Insights Into Antimicrobial Biosynthesis and Biocontrol Functions

The BGCs in strain 210 are associated with the production of putative of antimicrobial compounds similar to fusaricidin B, paenilan, tridecaptin, and paenicidin A. Fusaricidin B, for example, is active against *Fusarium oxysporum* and *Staphylococcus aureus* (Kajimura and Kaneda 1997; Lorentz et al. 2006; S. H. Lee et al. 2013; Xu et al. 2014; Jeong et al. 2019; Kim et al. 2020; Tsai et al. 2022; Yuan et al. 2022; Dobrzyński and Naziębło 2024). It is conserved across *Paenibacillus* spp., while paenilan production is uniquely shared with *P. polymyxa* (Jeong et al. 2019). Paenicidin A, targeting *L. monocytogenes* (Lorentz et al. 2006), and tridecaptin, effective against multidrug‐resistant Gram‐negative pathogens, highlight dual roles in environmental remediation and pathogen suppression. These antimicrobials mirror the biocontrol traits of *P. kribbensis* strain t‐9 (Xu et al. 2014), *P. peoriae* ZBSF16 (Yuan et al. 2022) and *P. polymyxa* E681 (Jeong et al. 2019) but expand strain 210's utility in agroindustrial applications, such as protecting crops from soil‐borne pathogens (Dobrzyński and Naziębło 2024).

Beyond the identification of individual gene clusters, the distribution of these BGCs reveals broader evolutionary and ecological dynamics within the genus (Supporting Information Table A4). The conservation of fusaricidin B, paeninodin, and tridecaptin clusters across closely related species points to a core antimicrobial repertoire essential for competitive fitness in soil environments (Supporting Information Figures A4–A6). In contrast, the more limited occurrence of paenilan and paenicidin likely reflects recent acquisition events or selective retention, highlighting the adaptive flexibility of *Paenibacillus* spp. (Supporting Information Figures A7 and A8). Collectively, these features position strain 210 not only as a taxonomically relevant isolate but also as a valuable reservoir of bioactive compounds with significant biotechnological potential.

### 4.5 Potential PGP Mechanisms and Agricultural Prospects of *Paenibacillus* sp. Strain 210

The genomic characterization of *Paenibacillus* sp. strain 210 suggests the presence of a robust suite of PGP mechanisms, positioning it as a formidable candidate for sustainable agricultural practices. The ability of strain 210 to potentially synthesize IAA, solubilize phosphate, and fix atmospheric nitrogen is inferred from its genomic repertoire, underscores its potential to enhance crop productivity while reducing reliance on synthetic fertilizers. These traits, identified through comparative genomics and supported by existing literature on *Paenibacillus* species, highlight strain 210's putative adaptability and ecological versatility.

In strain 210, IAA production, predicted to be mediated by genes such as *ipdC*, *trpA‐E* and *aroC/H/F*, presents metabolic pathways similar to those observed in *P. peoriae* ZBSF16, where it can also promote root elongation through auxin synthesis (Yuan et al. 2022). IAA is known to be critical for stimulating root architecture, thereby improving nutrient and water uptake in plants. While multiple *Paenibacillus* strains exhibit IAA production, strain 210 distinguishes itself through its chromosomal localization of these genes, as opposed to plasmid‐borne systems. This genomic stability may indicate a heritable and reliable trait, although experimental validation is required to confirm its functionality under environmental conditions.

The strain's predicted capacity to solubilize phosphate via *phoN*, *phoA*, and *phn/phs* operons aligns with the functional repertoire of *Paenibacillus kribensis* CX‐7, which efficiently converts insoluble phosphate into bioavailable forms (Ai‐min et al. 2013). The presence of this operon (Table 4), including regulatory and transport components such as *phoU*, suggests a coordinated system for phosphate acquisition and homeostasis under nutrient‐limited conditions, supporting the strain's putative phosphate‐solubilizing potential. In nutrient‐poor or polluted soils, such as those contaminated by hydrocarbons, these mechanisms could mitigate phosphorus limitation and contribute to plant growth promotion and soil recovery.

*Paenibacillus* sp. strain 210 *nif* gene cluster, encoding nitrogenase and FeMo‐cofactor biosynthesis proteins, indicates the potential for atmospheric nitrogen fixation—a trait shared with *P. brasilensis* PB24 (Do Carmo Dias et al. 2021). However, strain 210 further complements this with genomic evidence of nitrate reduction (*nar* operon) and ammonium transport (*amtB*), creating a dual nitrogen acquisition strategy. This genetically inferred metabolic flexibility suggests nitrogen availability across diverse soil conditions, from nitrogen‐depleted agricultural lands to hydrocarbon‐rich environments.

The combination of IAA production, phosphate solubilization, and nitrogen metabolism within a single strain reflects the multifunctionality observed in recently described species such as *Paenibacillus monticola* sp. nov., isolated from extreme environments (H. P. Li, Gan, et al. 2022). Notably, strain 210's genomic repertoire appears specifically adapted to thrive in crude oil–contaminated soils, an underexplored niche in plant growth‐promoting rhizobacteria (PGPR) research. Comparative analysis of its gene content with species exhibiting experimentally validated, desirable traits further supports the isolate's biotechnological potential (Supporting Information Table A8).

This adaptability suggests that strain 210 could potentially serve as a “pioneer” microbe in degraded ecosystems, simultaneously rehabilitating soils and promoting plant growth—a hypothesis that warrants experimental confirmation before application in commercial PGPR formulations.

## 5 Conclusion

*Paenibacillus* sp. 210 emerges as a versatile biocatalyst, combining genomic insights and in silico structural analyses to reveal its multifaceted potential. Its CAZyme repertoire and vitamin synthesis pathways support applications in soil revitalization, bioenergy production, and residue valorization (A. P. De Souza et al. 2014), while antimicrobial BGCs offer avenues for pathogen control in agriculture (Lorentz et al. 2006; Dobrzyński and Naziębło 2024). PGP mechanisms—including IAA production, phosphate solubilization, and nitrogen fixation—address critical agricultural challenges such as nutrient limitation, soil degradation, and pathogen pressure, providing a genetically resilient alternative to chemical inputs (Ai‐min et al. 2013; Do Carmo Dias et al. 2021; H. P. Li, Gan, et al. 2022). Our genomic analyses provide evidence supporting the potential for these capabilities. Comparative genomics further highlights the adaptability of strain 210 across bioenergy, agriculture, and environmental remediation sectors. However, further studies are required to experimentally evaluate plant–microorganism interactions, process stability, and practical applicability. Field trials in contaminated soils and exploration of synergies with hydrocarbon‐degrading consortia (Y. Li, Li, et al. 2022) could be important steps in this process. Overcoming challenges such as enzymatic scalability and consortium stability will be essential to fully exploit its metabolic flexibility for sustainable agriculture and circular bioeconomy initiatives. Overall, this study highlights the untapped potential of extremophilic isolates to drive ecological and agricultural innovation.

## Author Contributions

**João Victor dos Anjos Almeida:** conceptualization, methodology, investigation, analysis, writing – original draft, writing – review and editing. **Carlos Miguel Nóbrega Mendonça:** conceptualization, data collection, writing – review and editing. **Leandro Marcio Moreira:** methodology, writing – review and editing. **Ricardo Pinheiro de Souza Oliveira:** data collection, writing – review and editing, **Alessandro de Mello Varani:** conceptualization, methodology, supervision, writing – review and editing. **Mauro de Medeiros Oliveira:** conceptualization, methodology, conceptualization, data collection, methodology, investigation, analysis, supervision, writing – review and editing.

## Ethics Statement

The authors have nothing to report.

## Conflicts of Interest

The authors declare no conflicts of interest.

## Supporting information


**Figure A1:** Phylogenomic tree assembled using single‐copy genes shared between *Paenibacillus* reference genomes. In green highlight, the clade closest to *Paenibacillus* sp. used in comparative analyses.

**Figure A2:** Phylogenomic relationships within the clade containing *Paenibacillus* sp. strain 210, reconstructed with additional genomes. The analysis confirms its evolutionary placement.

**Figure A3:** Heatmap generated by ClusterANImap, showing the ANI index between genomes of *Paenibacillus* sp. and all reference genomes. Colors range from red (high similarity) to white (low similarity), with gray areas indicating the absence of a relationship. The dendrogram highlights phylogenetic clusters based on genomic similarity.

**Figure A4:** Synteny and collinearity relationships among *Paenibacillus* genomes, shared with *Paenibacillus* sp. strain 210 in regions associated with fusaricidin B biosynthesis. (paeninodin, tridecaptin, paenilan, paenicidin).

**Figure A5:** Synteny and collinearity relationships among *Paenibacillus* genomes, shared with *Paenibacillus* sp. strain 210 in regions associated with paeninodin biosynthesis.

**Figure A6:** Synteny and collinearity relationships among *Paenibacillus* genomes, shared with *Paenibacillus* sp. strain 210 in regions associated with tridecaptin biosynthesis.

**Figure A7:** Synteny and collinearity relationships among *Paenibacillus* genomes, shared with *Paenibacillus* sp. strain 210 in regions associated with paenilan biosynthesis.

**Figure A8:** Synteny and collinearity relationships among *Paenibacillus* genomes, shared with *Paenibacillus* sp. strain 210 in regions associated with paenicidin biosynthesis.

**Figure A9:** Ramachandran plot for the modeled enzymes involved in polysaccharide degradation pathways in strain 210, generated using SAVES 6.1. The plot illustrates the distribution of phi (ϕ) and psi (ψ) dihedral angles, highlighting the favored, allowed, and disallowed regions.

**Table A1:** Summary of reference genomes retrieved from the NCBI RefSeq database.

**Table A2:** Distribution of antibiotic resistance genes detected in genome analysis. Genomes that did not yield results for this analysis were not included in the table.

**Table A3:** Distribution of prophage regions detected in genome analysis. Genomes that did not yield results for this analysis were not included in the table.

**Table A4:** Results for regions linked to secondary metabolite production predicted by antiSMASH and BAGEL4.

**Table A5:** Distribution of CAZy terms detected in genome analysis.

**Table A6:** Metabolic pathway genes labeled and reconstructed by genomic profiling of strain 210.

**Table A7:** Labeled proteins and reference proteins used for comparisons in their primary and tertiary structure, by the predicted models.

**Table A8:** Distribution of genes related to plant growth promotion mechanisms in strain 210 and in the genomes of the closest species according to our phylogenomic results.

supmat.

## Data Availability

The genome assembly and annotations for *Paenibacillus* sp. strain 210 are available under the accession number CP160863, associated with BioProject PRJNA1121381 and BioSample SAMN41749029. Additional public genomic data utilized in this study are referenced throughout the manuscript and detailed in the supplementary materials.

## References

[mbo370159-bib-0001] Abramson, J. , J. Adler , J. Dunger , et al. 2024. “Accurate Structure Prediction of Biomolecular Interactions With Alphafold 3.” Nature 630, no. 8016: 493–500. 38718835 10.1038/s41586-024-07487-w PMC11168924

[mbo370159-bib-0002] Ai‐min, Z. , Z. Gang yong , G. Tong guo , et al. 2013. “Solubilization of Insoluble Potassium and Phosphate by *Paenibacillus kribensis* cx‐7: A Soil Microorganism With Biological Control Potential.” African Journal of Microbiology Research 7, no. 1: 41–47.

[mbo370159-bib-0003] Alcock, B. P. , W. Huynh , R. Chalil , et al. 2023. “CARD 2023: Expanded Curation, Support for Machine Learning, and Resistome Prediction at the Comprehensive Antibiotic Resistance Database.” Nucleic Acids Research 51, no. D1: D690–D699. 36263822 10.1093/nar/gkac920 PMC9825576

[mbo370159-bib-0004] Andreopoulos, W. B. , A. M. Geller , M. Lucke , et al. 2022. “Deeplasmid: Deep Learning Accurately Separates Plasmids From Bacterial Chromosomes.” Nucleic Acids Research 50, no. 3: e17. 34871418 10.1093/nar/gkab1115 PMC8860608

[mbo370159-bib-0005] Babalola, O. O. 2010. “Beneficial Bacteria of Agricultural Importance.” Biotechnology Letters 32: 1559–1570. 20635120 10.1007/s10529-010-0347-0

[mbo370159-bib-0006] Bervoets, I. , and D. Charlier . 2019. “Diversity, Versatility and Complexity of Bacterial Gene Regulation Mechanisms: Opportunities and Drawbacks for Applications in Synthetic Biology.” FEMS Microbiology Reviews 43, no. 3: 304–339. 30721976 10.1093/femsre/fuz001 PMC6524683

[mbo370159-bib-0007] Blin, K. , S. Shaw , H. E. Augustijn , et al. 2023. “antiSMASH 7.0: New and Improved Predictions for Detection, Regulation, Chemical Structures and Visualisation.” Nucleic Acids Research 51, no. W1: W46–W50. 37140036 10.1093/nar/gkad344 PMC10320115

[mbo370159-bib-0008] Bortolaia, V. , R. S. Kaas , E. Ruppe , et al. 2020. “ResFinder 4.0 for Predictions of Phenotypes From Genotypes.” Journal of Antimicrobial Chemotherapy 75, no. 12: 3491–3500. 32780112 10.1093/jac/dkaa345 PMC7662176

[mbo370159-bib-0009] Bouras, G. , S. R. Grigson , B. Papudeshi , V. Mallawaarachchi , and M. J. Roach . 2024. “DNAapler: A Tool to Reorient Circular Microbial Genomes.” Journal of Open Source Software 9, no. 93: 5968.

[mbo370159-bib-0010] Buchfink, B. , K. Reuter , and H. G. Drost . 2021. “Sensitive Protein Alignments at Tree‐of‐Life Scale Using DIAMOND.” Nature Methods 18: 366–368. 33828273 10.1038/s41592-021-01101-x PMC8026399

[mbo370159-bib-0011] Buntin, N. , T. Hongpattarakere , J. Ritari , et al. 2017. “An Inducible Operon Is Involved in Inulin Utilization in *Lactobacillus plantarum* Strains, as Revealed by Comparative Proteogenomics and Metabolic Profiling.” Applied and Environmental Microbiology 83, no. 2: e02402‐16. 27815279 10.1128/AEM.02402-16 PMC5203619

[mbo370159-bib-0012] Cantalapiedra, C. P. , A. Hernández‐Plaza , I. Letunic , P. Bork , and J. Huerta‐Cepas . 2021. “eggNOG‐Mapper v2: Functional Annotation, Orthology Assignments, and Domain Prediction at the Metagenomic Scale.” Molecular Biology and Evolution 38, no. 12: 5825–5829. 34597405 10.1093/molbev/msab293 PMC8662613

[mbo370159-bib-0013] Carattoli, A. , E. Zankari , A. García‐Fernández , et al. 2014. “In Silico Detection and Typing of Plasmids Using Plasmidfinder and Plasmid Multilocus Sequence Typing.” Antimicrobial Agents and Chemotherapy 58, no. 7: 3895–3903. 24777092 10.1128/AAC.02412-14 PMC4068535

[mbo370159-bib-0014] Chaumeil, P. A. , A. J. Mussig , P. Hugenholtz , and D. H. Parks . 2022. “GTDB‐Tk v2: Memory Friendly Classification With the Genome Taxonomy Database.” Bioinformatics 38, no. 23: 5315–5316. 36218463 10.1093/bioinformatics/btac672 PMC9710552

[mbo370159-bib-0015] Chun, J. , A. Oren , A. Ventosa , et al. 2018. “Proposed Minimal Standards for the Use of Genome Data for the Taxonomy of Prokaryotes.” International Journal of Systematic and Evolutionary Microbiology 68, no. 1: 461–466. 29292687 10.1099/ijsem.0.002516

[mbo370159-bib-0016] Cumsille, A. , R. E. Durán , A. Rodríguez‐Delherbe , et al. 2023. “GenoVi, an Open‐Source Automated Circular Genome Visualizer for Bacteria and Archaea.” PLoS Computational Biology 19, no. 4: e1010998. 37014908 10.1371/journal.pcbi.1010998 PMC10104344

[mbo370159-bib-0017] Da Mota, F. F. , E. A. Gomes , E. Paiva , A. S. Rosado , and L. Seldin . 2004. “Use of *rpoB* Gene Analysis for Identification of Nitrogen‐Fixing *Paenibacillus* Species as an Alternative to the 16s rRNA Gene.” Letters in Applied Microbiology 39, no. 1: 34–40. 15189285 10.1111/j.1472-765X.2004.01536.x

[mbo370159-bib-0018] Da Silva, M. B. F. , E. A. Lemos , R. E. Vollú , F. Abreu , A. S. Rosado , and L. Seldin . 2022. “ *Paenibacillus piscarius* sp. nov., a Novel Nitrogen‐Fixing Species Isolated From the Gut of the Armored Catfish *Parotocinclus maculicauda* .” Antonie Van Leeuwenhoek 115, no. 1: 155–165. 34993761 10.1007/s10482-021-01694-5

[mbo370159-bib-0019] De Souza, A. P. , A. Grandis , D. C. C. Leite , and M. S. Buckeridge . 2014. “Sugarcane as a Bioenergy Source: History, Performance, and Perspectives for Second‐Generation Bioethanol.” BioEnergy Research 7: 24–35.

[mbo370159-bib-0020] De Souza, A. P. , D. C. C. Leite , S. Pattathil , M. G. Hahn , and M. S. Buckeridge . 2013. “Composition and Structure of Sugarcane Cell Wall Polysaccharides: Implications for Second‐Generation Bioethanol Production.” BioEnergy Research 6: 564–579.

[mbo370159-bib-0021] De Souza, W. R. 2013. “Microbial Degradation of Lignocellulosic Biomass.” Sustainable Degradation of Lignocellulosic Biomass—Techniques, Applications and Commercialization 15: 207–247.

[mbo370159-bib-0022] Dewiyanti, I. , D. Darmawi , Z. A. Muchlisin , and T. Z. Helmi . 2024. “Analyzing Cellulolytic Bacteria Diversity in Mangrove Ecosystem Soil Using 16 Svedberg Ribosomal Ribonucleic Acid Gene.” Global Journal of Environmental Science and Management 10, no. 1: 51–68.

[mbo370159-bib-0023] Dobrzyński, J. , and A. Naziębło . 2024. “ *Paenibacillus* as a Biocontrol Agent for Fungal Phytopathogens: Is *P. polymyxa* the Only One Worth Attention?” Microbial Ecology 87, no. 1: 134. 39480531 10.1007/s00248-024-02450-8 PMC11527970

[mbo370159-bib-0024] Do Carmo Dias, B. , F. F. da Mota , D. Jurelevicius , and L. Seldin . 2021. “Genetics and Regulation of Nitrogen Fixation in *Paenibacillus brasilensis* PB24.” Microbiological Research 243: 126647. 33290933 10.1016/j.micres.2020.126647

[mbo370159-bib-0025] Doster, E. , S. M. Lakin , C. J. Dean , et al. 2020. “MEGARES 2.0: A Database for Classification of Antimicrobial Drug, Biocide and Metal Resistance Determinants in Metagenomic Sequence Data.” Nucleic Acids Research 48, no. D1: D561–D569. 31722416 10.1093/nar/gkz1010 PMC7145535

[mbo370159-bib-0026] Drula, E. , M. L. Garron , S. Dogan , V. Lombard , B. Henrissat , and N. Terrapon . 2022. “The Carbohydrate‐Active Enzyme Database: Functions and Literature.” Nucleic Acids Research 50, no. D1: D571–D577. 34850161 10.1093/nar/gkab1045 PMC8728194

[mbo370159-bib-0027] Egorov, A. A. , and G. C. Atkinson . 2025. “LoVis4u: A Locus Visualization Tool for Comparative Genomics and Coverage Profiles.” NAR Genomics and Bioinformatics 7, no. 1: lqaf009. 40007724 10.1093/nargab/lqaf009 PMC11850299

[mbo370159-bib-0028] Feldgarden, M. , V. Brover , N. Gonzalez‐Escalona , et al. 2021. “AMRFinderPlus and the Reference Gene Catalog Facilitate Examination of the Genomic Links Among Antimicrobial Resistance, Stress Response, and Virulence.” Scientific Reports 11: 12728. 34135355 10.1038/s41598-021-91456-0 PMC8208984

[mbo370159-bib-0029] Feng, X. , H. Cheng , D. Portik , and H. Li . 2022. “Metagenome Assembly of High‐Fidelity Long Reads With Hifiasm‐Meta.” Nature Methods 19, no. 6: 671–674. 35534630 10.1038/s41592-022-01478-3 PMC9343089

[mbo370159-bib-0030] Grady, E. N. , J. MacDonald , L. Liu , A. Richman , and Z. C. Yuan . 2016. “Current Knowledge and Perspectives of *Paenibacillus*: A Review.” Microbial Cell Factories 15, no. 1: 203. 27905924 10.1186/s12934-016-0603-7 PMC5134293

[mbo370159-bib-0031] Guo, B. , S. Dai , R. Wang , J. Guo , Y. Ding , and Y. Xu . 2015. “Combined Effects of Elevated CO_2_ and Cd‐Contaminated Soil on the Growth, Gas Exchange, Antioxidant Defense, and Cd Accumulation of Poplars and Willows.” Environmental and Experimental Botany 115: 1–10.

[mbo370159-bib-0032] Gupta, S. K. , B. R. Padmanabhan , S. M. Diene , et al. 2014. “Arg‐Annot, a New Bioinformatic Tool to Discover Antibiotic Resistance Genes in Bacterial Genomes.” Antimicrobial Agents and Chemotherapy 58, no. 1: 212–220. 24145532 10.1128/AAC.01310-13 PMC3910750

[mbo370159-bib-0033] He, M. X. , Y. Li , X. Liu , F. Bai , H. Feng , and Y. Z. Zhang . 2009. “Ethanol Production by Mixed‐Cultures of *Paenibacillus* sp. and *Zymomonas mobilis* Using the Raw Starchy Material From Sweet Potato.” Annals of Microbiology 59: 749–754.

[mbo370159-bib-0034] Jang, H. J. , S. H. Yoon , H. K. Ryu , et al. 2011. “Retinoid Production Using Metabolically Engineered *Escherichia coli* With a Two‐Phase Culture System.” Microbial Cell Factories 10: 59. 21801353 10.1186/1475-2859-10-59 PMC3160355

[mbo370159-bib-0035] Jeong, H. , S. K. Choi , C. M. Ryu , and S. H. Park . 2019. “Chronicle of a Soil Bacterium: *Paenibacillus polymyxa* E681 as a Tiny Guardian of Plant and Human Health.” Frontiers in Microbiology 10: 467. 30930873 10.3389/fmicb.2019.00467 PMC6429003

[mbo370159-bib-0036] Jinfang, Z. , Q. Ge , Y. Yan , X. Zhang , L. Huang , and Y. Yin . 2023. “dbCAN3: Automated Carbohydrate‐Active Enzyme and Substrate Annotation.” Nucleic Acids Research 51, no. W1: 115–121. 10.1093/nar/gkad328 PMC10320055 37125649

[mbo370159-bib-0037] Jones, P. , D. Binns , H.‐Y. Chang , et al. 2014. “InterProScan 5: Genome‐Scale Protein Function Classification.” Bioinformatics 30, no. 9: 1236–1240. 24451626 10.1093/bioinformatics/btu031 PMC3998142

[mbo370159-bib-0038] Jonsson, A. P. , and T. L. Östberg . 2011. “The Effects of Carbon Sources and Micronutrients in Whey and Fermented Whey on the Kinetics of Phenanthrene Biodegradation in Diesel‐Contaminated Soil.” Journal of Hazardous Materials 192, no. 3: 1171–1177. 21741168 10.1016/j.jhazmat.2011.06.024

[mbo370159-bib-0039] Kajimura, Y. , and M. Kaneda . 1997. “Fusaricidins B, C and D, New Depsipeptide Antibuotics Produced by *Bacillus polymyxa* KT‐8: Isolation, Structure Elucidation and Biological Activity.” Journal of Antibiotics 50, no. 3: 220–228. 9439693

[mbo370159-bib-0040] Kalyaanamoorthy, S. , B. Q. Minh , T. K. F. Wong , A. Von Haeseler , and L. S. Jermiin . 2017. “ModelFinder: Fast Model Selection for Accurate Phylogenetic Estimates.” Nature Methods 14, no. 6: 587–589. 28481363 10.1038/nmeth.4285 PMC5453245

[mbo370159-bib-0041] Kanehisa, M. 2000. “KEGG: Kyoto Encyclopedia of Genes and Genomes.” Nucleic Acids Research 28, no. 1: 27–30. 10592173 10.1093/nar/28.1.27 PMC102409

[mbo370159-bib-0042] Kim, J. , K. D. Le , N. H. Yu , J. I. Kim , J. C. Kim , and C. W. Lee . 2020. “Structure and Antifungal Activity of Pelgipeptins From *Paenibacillus elgii* Against Phytopathogenic Fungi.” Pesticide Biochemistry and Physiology 163: 154–163. 31973853 10.1016/j.pestbp.2019.11.009

[mbo370159-bib-0043] Kong, J. , Z. Fu , Y. Liu , et al. 2025. “ *Paenibacillus hubeiensis* sp. nov.: A Novel Selenium‐Resistant Bacterium Isolated From the Rhizosphere of *Galinsoga parviflora* in a Selenium‐Rich Region of Enshi, Hubei Province.” Microorganisms 13, no. 7: 1559. 40732068 10.3390/microorganisms13071559 PMC12300063

[mbo370159-bib-0044] Lee, I. , Y. Ouk Kim , S. C. Park , and J. Chun . 2016. “OrthoANI: An Improved Algorithm and Software for Calculating Average Nucleotide Identity.” International Journal of Systematic and Evolutionary Microbiology 66, no. 2: 1100–1103. 26585518 10.1099/ijsem.0.000760

[mbo370159-bib-0045] Lee, S. H. , Y. E. Cho , S. H. Park , et al. 2013. “An Antibiotic Fusaricidin: A Cyclic Depsipeptide From *Paenibacillus polymyxa* E681 Induces Systemic Resistance Against *Phytophthora* Blight of Red‐Pepper.” Phytoparasitica 41: 49–58.

[mbo370159-bib-0046] Li, H. P. , Y. N. Gan , L. J. Yue , et al. 2022. “Newly Isolated *Paenibacillus monticola* sp. nov., a Novel Plant Growth‐Promoting Rhizobacteria Strain From High‐Altitude Spruce Forests in the Qilianmountains, China.” Frontiers in Microbiology 13: 833313. 35250949 10.3389/fmicb.2022.833313 PMC8895201

[mbo370159-bib-0047] Li, Z. , L. Jaroszewski , M. Iyer , M. Sedova , and A. Godzik . 2020. “FATCAT 2.0: Towards a Better Understanding of the Structural Diversity of Proteins.” Nucleic Acids Research 48, no. W1: W60–W64. 32469061 10.1093/nar/gkaa443 PMC7319568

[mbo370159-bib-0048] Li, Y. , C. Li , Y. Xin , T. Huang , and J. Liu . 2022. “Petroleum Pollution Affects Soil Chemistry and Reshapes the Diversity and Networks of Microbial Communities.” Ecotoxicology and Environmental Safety 246: 114129. 36193589 10.1016/j.ecoenv.2022.114129

[mbo370159-bib-0049] Li, T. , S. Wang , C. Liu , Y. Yu , M. Zong , and C. Duan . 2024. “Soil Microbial Communities' Contributions to Soil Ecosystem Multifunctionality in the Natural Restorationof Abandoned Metal Mines.” Journal of Environmental Management 353: 120244. 38335599 10.1016/j.jenvman.2024.120244

[mbo370159-bib-0050] Lohans, C. T. , Z. Huang , M. J. van Belkum , et al. 2012. “Structural Characterization of the Highly Cyclized Lantibiotic Paenicidin A via a Partial Desulfurization/Reduction Strategy.” Journal of the American Chemical Society 134, no. 48: 19540–19543. 23167271 10.1021/ja3089229

[mbo370159-bib-0051] Lorentz, R. H. , S. Ártico , A. B. Da Silveira , A. Einsfeld , and G. Corção . 2006. “Evaluation of Antimicrobial Activity in *Paenibacillus* spp. Strains Isolated From Natural Environment.” Letters in Applied Microbiology 43, no. 5: 541–547. 17032229 10.1111/j.1472-765X.2006.01995.x

[mbo370159-bib-0052] Luo, S. , J. Zhao , Y. Zheng , T. Chen , and Z. Wang . 2023. “Biosynthesis of Nicotinamide Mononucleotide: Current Metabolic Engineering Strategies, Challenges, and Prospects.” Fermentation 9, no. 7: 594.

[mbo370159-bib-0053] Mendonça, C. M. N. , R. C. Oliveira , R. K. B. Freire , et al. 2021. “Characterization of Levan Produced by a *Paenibacillus* sp. Isolated From Brazilian Crude Oil.” International Journal of Biological Macromolecules 186, no. 5: 788–799. 34245738 10.1016/j.ijbiomac.2021.07.036

[mbo370159-bib-0054] Minh, B. Q. , H. A. Schmidt , O. Chernomor , et al. 2020. “IQ‐TREE 2: New Models and Efficient Methods for Phylogenetic Inference in the Genomic Era.” Molecular Biology and Evolution 37, no. 5: 1530–1534. 32011700 10.1093/molbev/msaa015 PMC7182206

[mbo370159-bib-0055] Overbeek, R. , R. Olson , G. D. Pusch , et al. 2014. “The SEED and the Rapid Annotation of Microbial Genomes Using Subsystems Technology (RAST).” Nucleic Acids Research 42, no. D1: D206–D214. 24293654 10.1093/nar/gkt1226 PMC3965101

[mbo370159-bib-0056] Paës, G. , J. G. Berrin , and J. Beaugrand . 2012. “GH11 Xylanases: Structure/Function/Properties Relationships and Applications.” Biotechnology Advances 30, no. 3: 564–592. 22067746 10.1016/j.biotechadv.2011.10.003

[mbo370159-bib-0057] Pagliuso, D. , A. Grandis , C. R. De Sousa , A. P. de Souza , C. Driemeier , and M. S. Buckeridge . 2021. “The Effect of Sugarcane Straw Aging in the Field on Cell Wall Composition.” Frontiers in Plant Science 12: 652168. 34335640 10.3389/fpls.2021.652168 PMC8319731

[mbo370159-bib-0058] Parks, D. H. , M. Imelfort , C. T. Skennerton , P. Hugenholtz , and G. W. Tyson . 2015. “CheckM: Assessing the Quality of Microbial Genomes Recovered From Isolates, Single Cells, and Metagenomes.” Genome Research 25, no. 7: 1043–1055. 25977477 10.1101/gr.186072.114 PMC4484387

[mbo370159-bib-0059] Polyak, Y. M. , L. G. Bakina , N. V. Mayachkina , et al. 2024. “Long‐Term Effects of Oil Contamination on Soil Quality and Metabolic Function.” Environmental Geochemistry and Health 46, no. 1: 13. 10.1007/s10653-023-01779-2 38147148

[mbo370159-bib-0060] Potter, S. C. , A. Luciani , S. R. Eddy , Y. Park , R. Lopez , and R. D. Finn . 2018. “HMMER Web Server: 2018 Update.” Nucleic Acids Research 46, no. W1: W200–W204. 29905871 10.1093/nar/gky448 PMC6030962

[mbo370159-bib-0061] Radice, R. P. , V. De Fabrizio , A. Donadoni , A. Scopa , and G. Martelli . 2023. “Crude Oil Bioremediation: From Bacteria to Microalgae.” Processes 11, no. 2: 442.

[mbo370159-bib-0062] Sichert, A. , and O. X. Cordero . 2021. “Polysaccharide‐Bacteria Interactions From the Lens of Evolutionary Ecology.” Frontiers in Microbiology 12: 705082. 34690949 10.3389/fmicb.2021.705082 PMC8531407

[mbo370159-bib-0063] Simão, F. A. , R. M. Waterhouse , P. Ioannidis , E. V. Kriventseva , and E. M. Zdobnov . 2015. “BUSCO: Assessing Genome Assembly and Annotation Completeness With Single‐Copy or Thologs.” Bioinformatics 31, no. 19: 3210–3212. 26059717 10.1093/bioinformatics/btv351

[mbo370159-bib-0064] Sinha, A. K. , M. F. Laursen , and T. R. Licht . 2025. “Regulation of Microbial Gene Expression: The Key to Understanding Our Gut Microbiome.” Trends in Microbiology 33, no. 4: 397–407. 39095208 10.1016/j.tim.2024.07.005

[mbo370159-bib-0065] Tang, X. , J. Shang , Y. Ji , and Y. Sun . 2023. “PLASMe: A Tool to Identify PLASMid Contigs From Short‐Read Assemblies Using Transformer.” Nucleic Acids Research 51, no. 15: e83. 37427782 10.1093/nar/gkad578 PMC10450166

[mbo370159-bib-0066] Tatusova, T. , M. DiCuccio , A. Badretdin , et al. 2016. “NCBI Prokaryotic Genome Annotation Pipeline.” Nucleic Acids Research 44, no. 14: 6614–6624. 27342282 10.1093/nar/gkw569 PMC5001611

[mbo370159-bib-0067] Teufel, F. , J. J. Almagro Armenteros , A. R. Johansen , et al. 2022. “Signalp 6.0 Predicts All Five Types of Signal Peptides Using Protein Language Models.” Nature Biotechnology 40, no. 7: 1023–1025. 10.1038/s41587-021-01156-3 PMC9287161 34980915

[mbo370159-bib-0068] Tsai, S. H. , Y. T. Chen , Y. L. Yang , B. Y. Lee , C. J. Huang , and C. Y. Chen . 2022. “The Potential Biocontrol Agent *Paenibacillus polymyxa* TP3 Produces Fusaricidin‐Type Compounds Involved in the Antagonism Against Gray Mold Pathogen *Botrytis cinerea* .” Phytopathology 112: 775–783. 34587815 10.1094/PHYTO-04-21-0178-R

[mbo370159-bib-0069] Uebanso, T. , T. Shimohata , K. Mawatari , and A. Takahashi . 2020. “Functional Roles of B‐Vitamins in the Gut and Gut Microbiome.” Molecular Nutrition & Food Research 64, no. 18: 2000426. 10.1002/mnfr.202000426 32761878

[mbo370159-bib-0070] van Heel, A. J. , A. de Jong , C. Song , J. H. Viel , J. Kok , and O. P. Kuipers . 2018. “BAGEL4: A User‐Friendly Web Server to Thoroughly Mine Ripps and Bacteriocin.” Nucleic Acids Research 46, no. W1: 278–281. 10.1093/nar/gky383 PMC6030817 29788290

[mbo370159-bib-0071] Wang, M. , Y. Lian , Y. Wang , and L. Zhu . 2023. “The Role and Mechanism of Quorum Sensing on Environmental Antimicrobial Resistance.” Environmental Pollution 322: 121238. 36758922 10.1016/j.envpol.2023.121238

[mbo370159-bib-0072] Watanabe, S. , T. Kodak , and K. Makino . 2006. “Cloning, Expression, and Characterization of Bacterial l‐Arabinose 1‐Dehydrogenase Involved in an Alternative Pathway of l‐Arabinose Metabolism.” Journal of Biological Chemistry 281, no. 5: 2612–2623. 16326697 10.1074/jbc.M506477200

[mbo370159-bib-0073] Wishart, D. S. , S. Han , S. Saha , et al. 2023. “PHASTEST: Faster Than PHASTER, Better Than Phast.” Nucleic Acids Research 51, no. W1: W443–W450. 37194694 10.1093/nar/gkad382 PMC10320120

[mbo370159-bib-0074] Xu, S. J. , S. J. Hong , W. Choi , and B. S. Kim . 2014. “Antifungal Activity of *Paenibacillus kribbensis* Strain t‐9 Isolated From Soils Against Several Plant Pathogenic Fungi.” Plant Pathology Journal 30, no. 1: 102–108. 25288992 10.5423/PPJ.OA.05.2013.0052 PMC4174836

[mbo370159-bib-0075] Xue, H. , Y. Tu , T. Ma , N. Jiang , C. Piao , and Y. Li . 2023. “Taxonomic Study of Three Novel *Paenibacillus* Species With Cold‐Adapted Plant Growth‐Promoting Capacities Isolated From Root of *Larix gmelinii* .” Microorganisms 11, no. 1: 130. 36677422 10.3390/microorganisms11010130 PMC9867441

[mbo370159-bib-0076] Yuan, L. , H. Jiang , X. Jiang , et al. 2022. “Comparative Genomic and Functional Analyses of *Paenibacillus peoriae* ZBSF16 With Biocontrol Potential Against Grapevine Diseases, Provide Insights into Its Genes Related to Plant Growth‐Promoting and Biocontrol Mechanisms.” Frontiers in Microbiology 13: 975344. 36160187 10.3389/fmicb.2022.975344 PMC9492885

[mbo370159-bib-0077] Zabed, H. , J. N. Sahu , A. Suely , A. N. Boyce , and G. Faruq . 2017. “Bioethanol Production From Renewable Sources: Current Perspectives and Technological Progress.” Renewable and Sustainable Energy Reviews 71: 475–501.

