# Supplementary figures and images for: Genome‐Driven Analysis Reveals the Biotechnological Potential of a Novel Paenibacillus sp. Isolated From Crude Oil
Source: Microbiologyopen. 2025 Nov 24;14(6):e70159. doi: 10.1002/mbo3.70159 (PMC12643537; doi:10.1002/mbo3.70159)

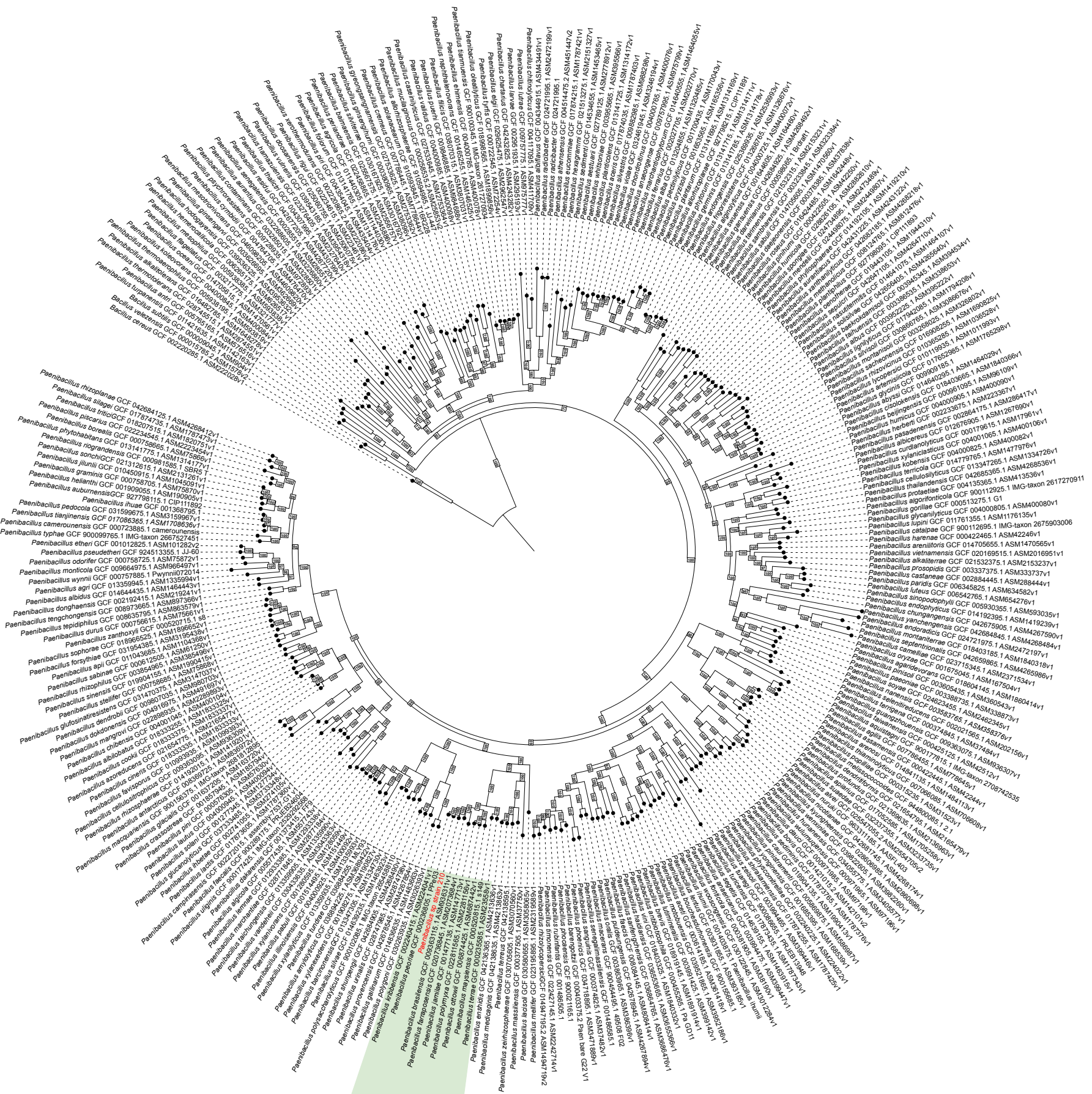

Supplement: Supplementary file 1 — Figure A1: Phylogenomic tree assembled using single‐copy genes shared between Paenibacillus reference genomes. In green highlight, the clade closest to Paenibacillus sp. used in comparative analyses. [file MBO3-14-e70159-s005.pdf]

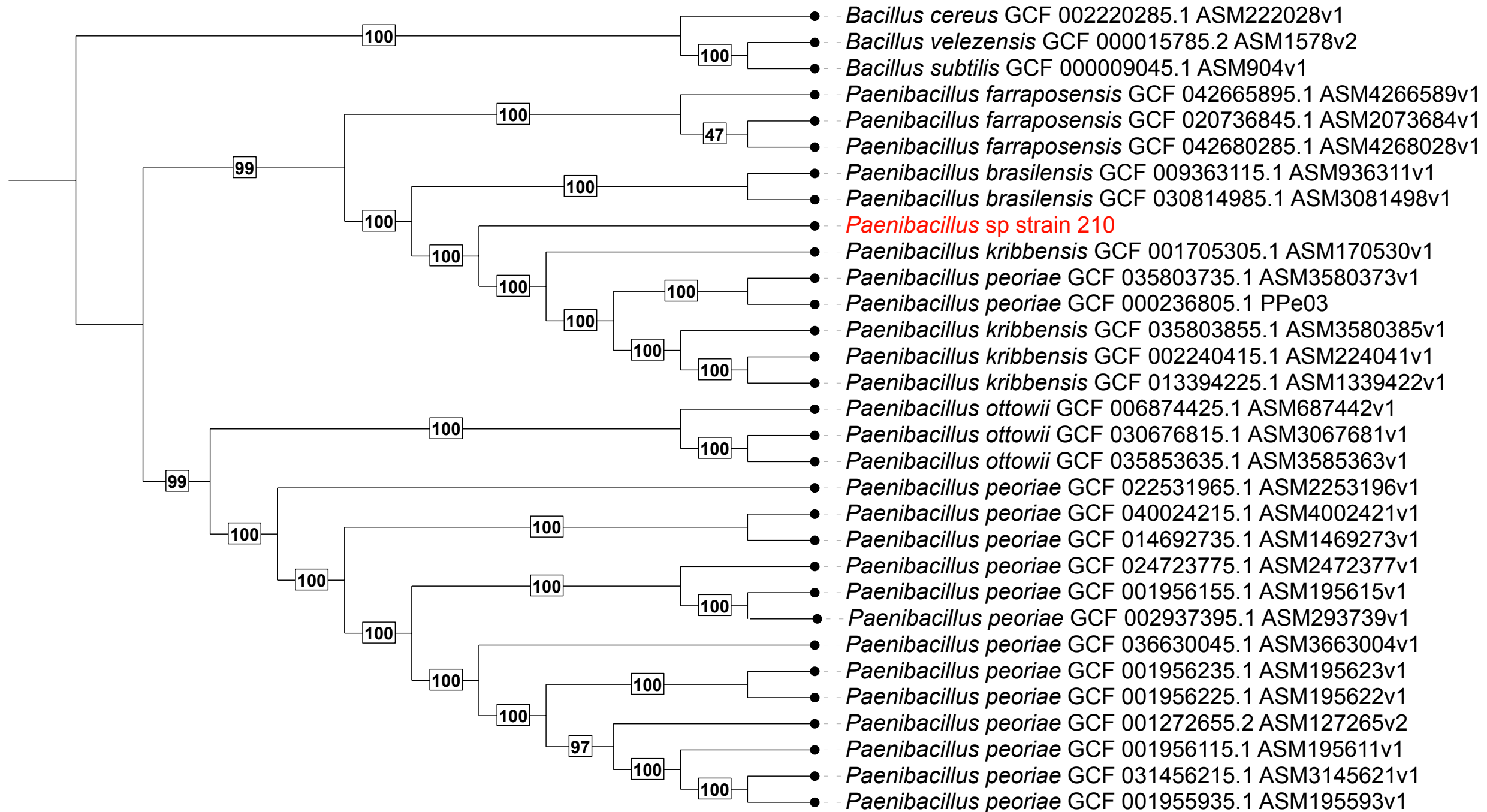

Supplement: Supplementary file 2 — Figure A2: Phylogenomic relationships within the clade containing Paenibacillus sp. strain 210, reconstructed with additional genomes. The analysis confirms its evolutionary placement. [file MBO3-14-e70159-s008.pdf]

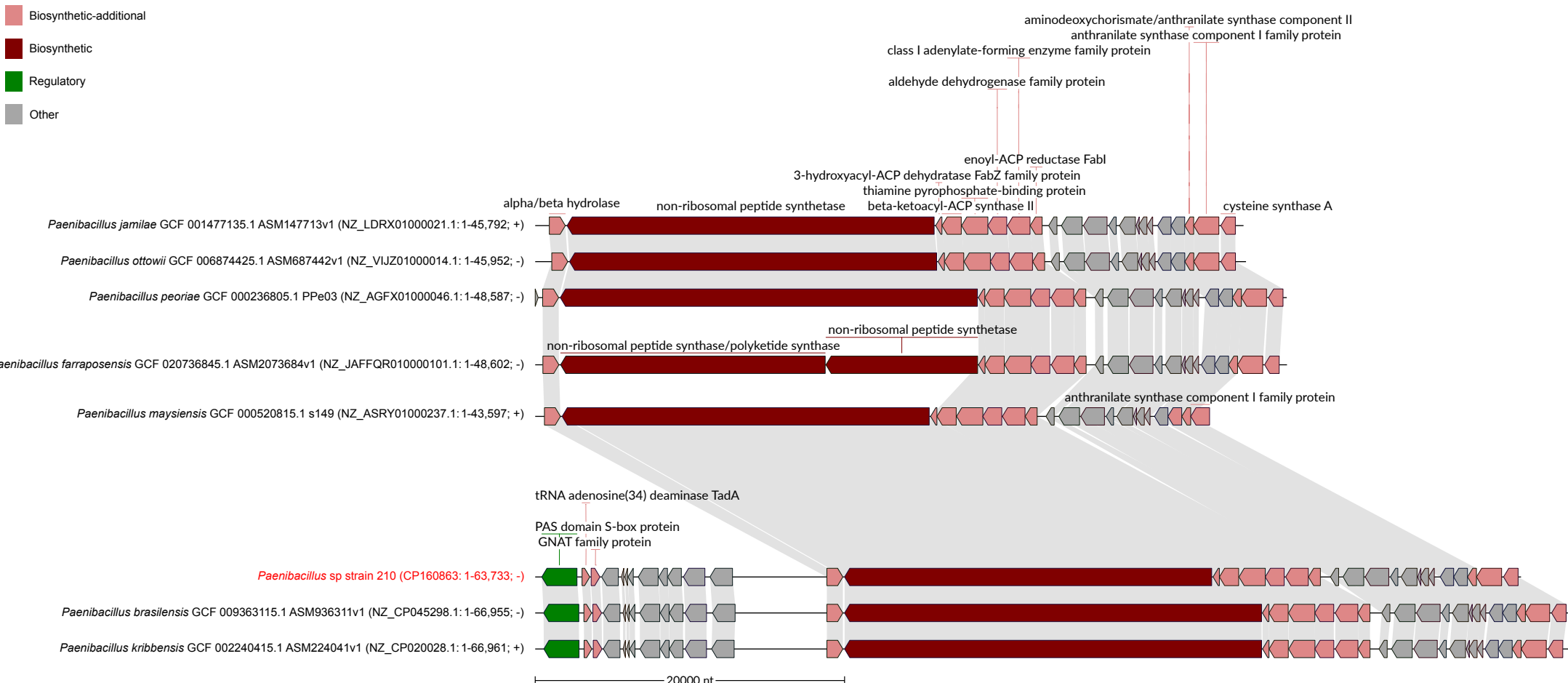

Supplement: Supplementary file 4 — Figure A4: Synteny and collinearity relationships among Paenibacillus genomes, shared with Paenibacillus sp. strain 210 in regions associated with fusaricidin B biosynthesis. (paeninodin, tridecaptin, paenilan, paenicidin). [file MBO3-14-e70159-s017.pdf]

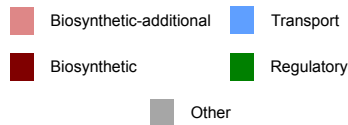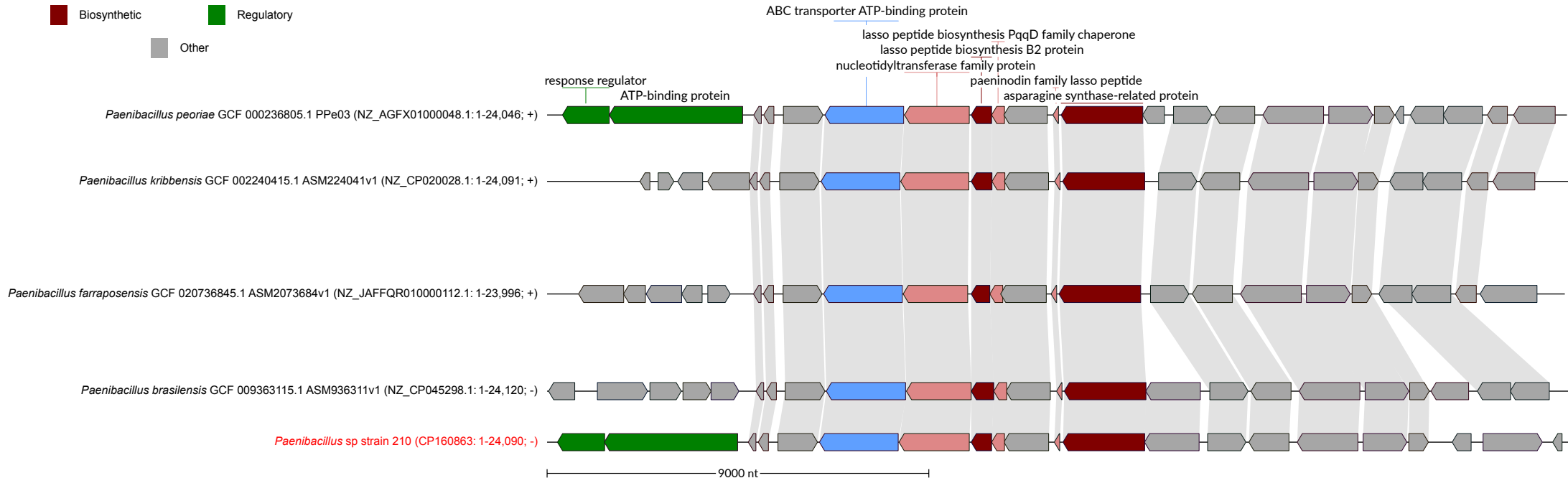

Supplement: Supplementary file 5 — Figure A5: Synteny and collinearity relationships among Paenibacillus genomes, shared with Paenibacillus sp. strain 210 in regions associated with paeninodin biosynthesis. [file MBO3-14-e70159-s011.pdf]

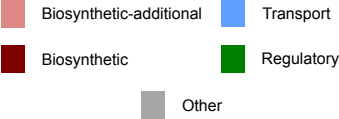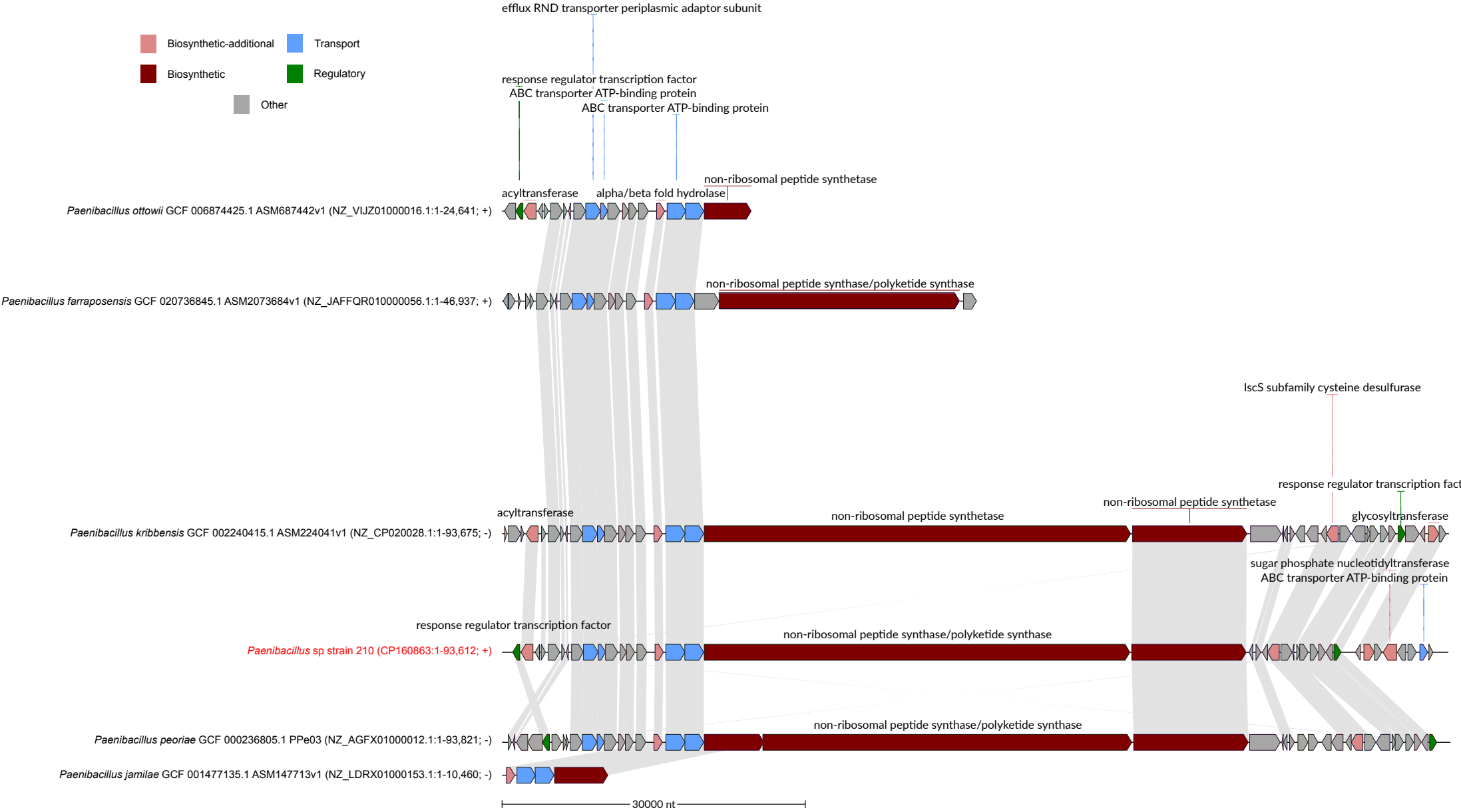

Supplement: Supplementary file 6 — Figure A6: Synteny and collinearity relationships among Paenibacillus genomes, shared with Paenibacillus sp. strain 210 in regions associated with tridecaptin biosynthesis. [file MBO3-14-e70159-s010.pdf]

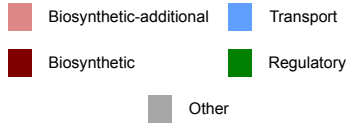

*Paenibacillus* sp strain 210 (CP160863:1-26,451; +)

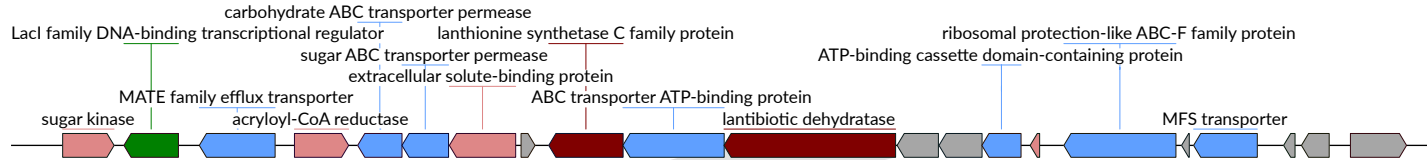

*Paenibacillus tianmuensis* GCF 900100345.1 IMG-taxon 2617270934 (NZ\_FMTT01000007.1:1-14,236; -)

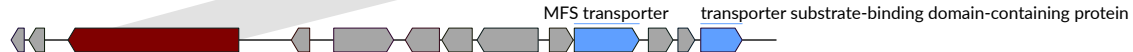

10000 nt

Supplement: Supplementary file 8 — Figure A8: Synteny and collinearity relationships among Paenibacillus genomes, shared with Paenibacillus sp. strain 210 in regions associated with paenicidin biosynthesis. [file MBO3-14-e70159-s016.pdf]

### 3.2.1.4

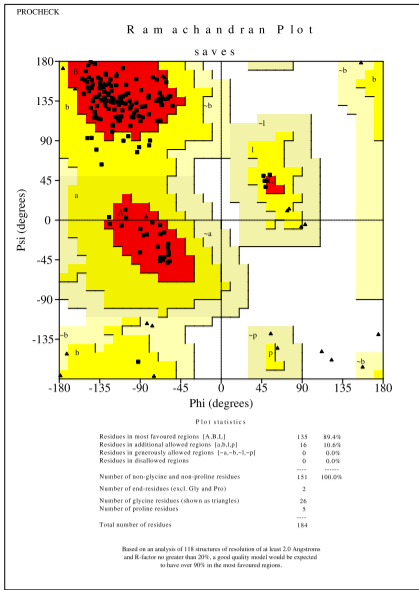

AB1387\_04845

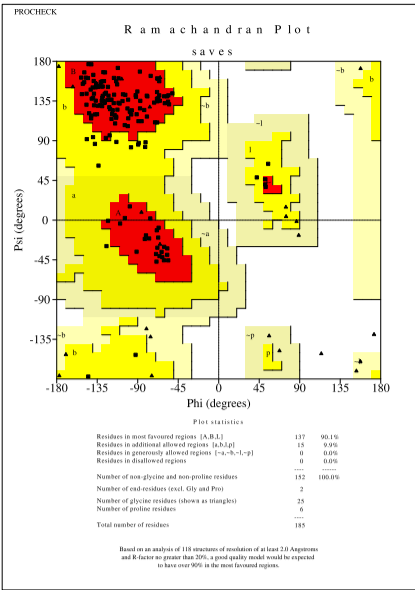

P18429

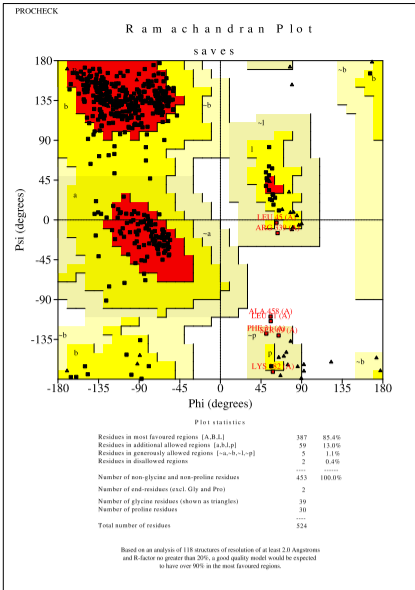

AB1387\_16250

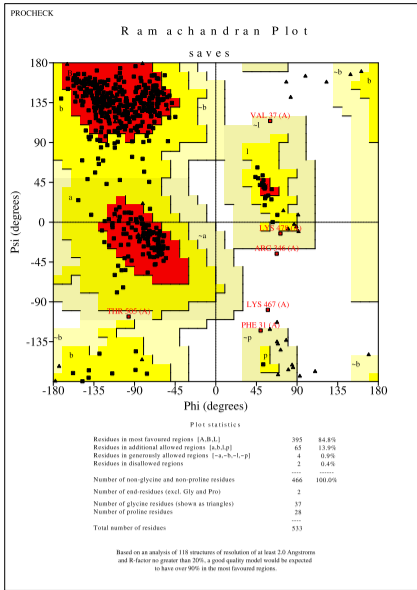

P94489

### 2.7.1.17

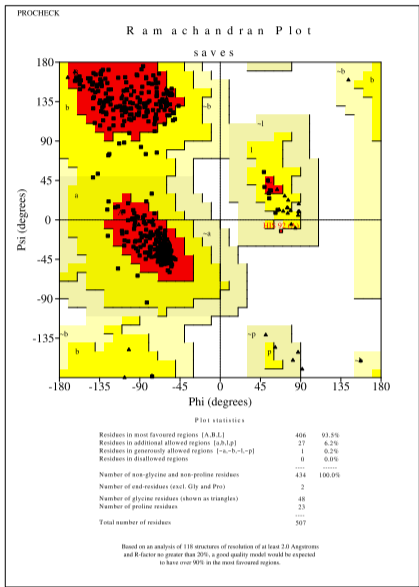

AB1387 21475

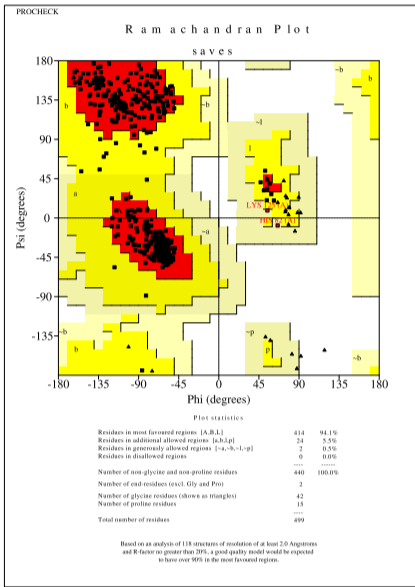

P39211

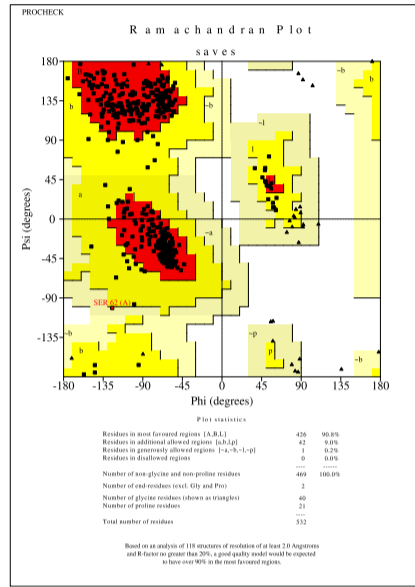

AB1387\_25700

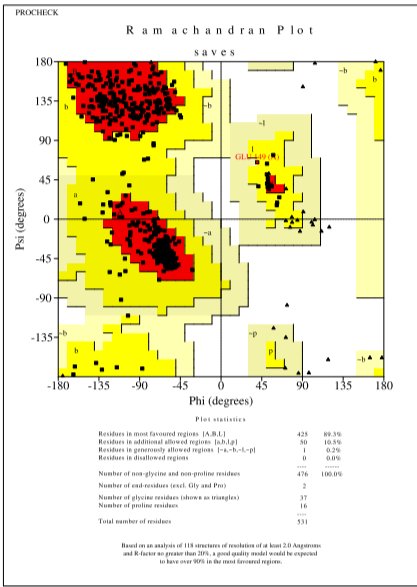

D4P8C6

### 3.2.1.4

### 3.2.1.21

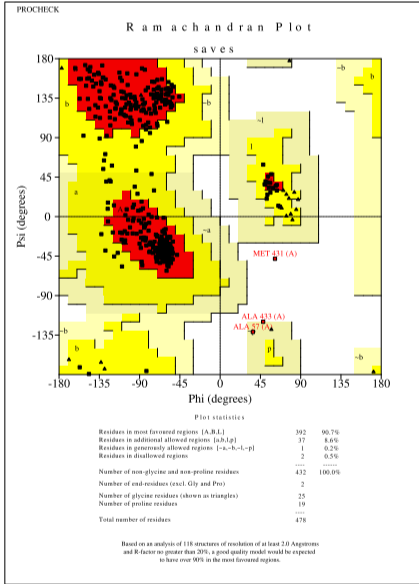

AB1387 03255

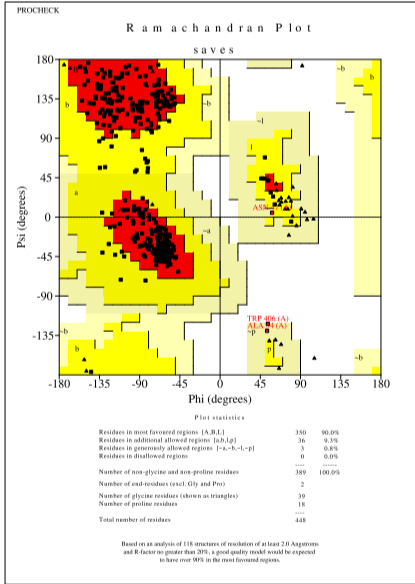

P22073

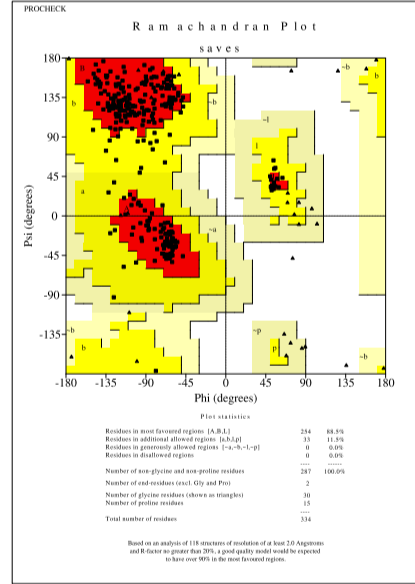

AB1387 16255

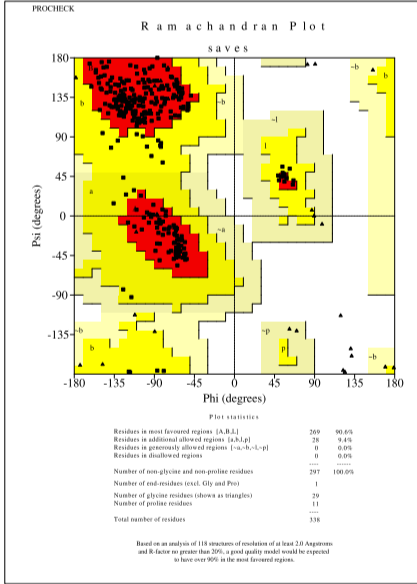

# POC1A9

### 3.1.1.11

### 3.2.1.67

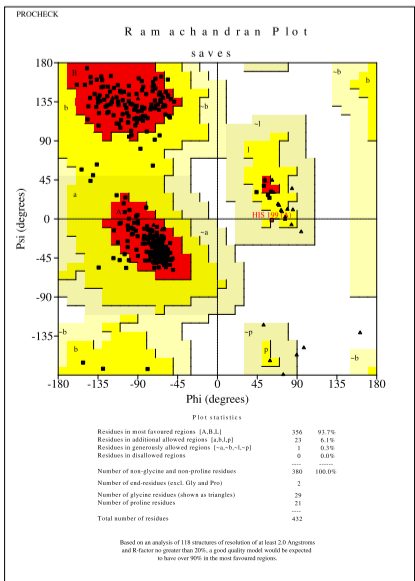

AB1387 04930

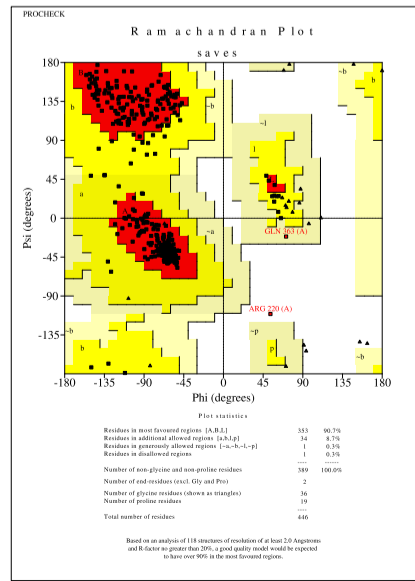

P39130

Supplement: Supplementary file 9 — Figure A9: Ramachandran plot for the modeled enzymes involved in polysaccharide degradation pathways in strain 210, generated using SAVES 6.1. The plot illustrates the distribution of phi (ϕ) and psi (ψ) dihedral angles, highlighting the favored, allowed, and disallowed regions. [file MBO3-14-e70159-s006.pdf]
