# Supplementary material for: Genome‐Driven Analysis Reveals the Biotechnological Potential of a Novel Paenibacillus sp. Isolated From Crude Oil
Source: Microbiologyopen. 2025 Nov 24;14(6):e70159. doi: 10.1002/mbo3.70159 (PMC12643537; doi:10.1002/mbo3.70159)
Supplement: Supplementary file 7 — Figure A7: Synteny and collinearity relationships among Paenibacillus genomes, shared with Paenibacillus sp. strain 210 in regions associated with paenilan biosynthesis. [file MBO3-14-e70159-s001.pdf]

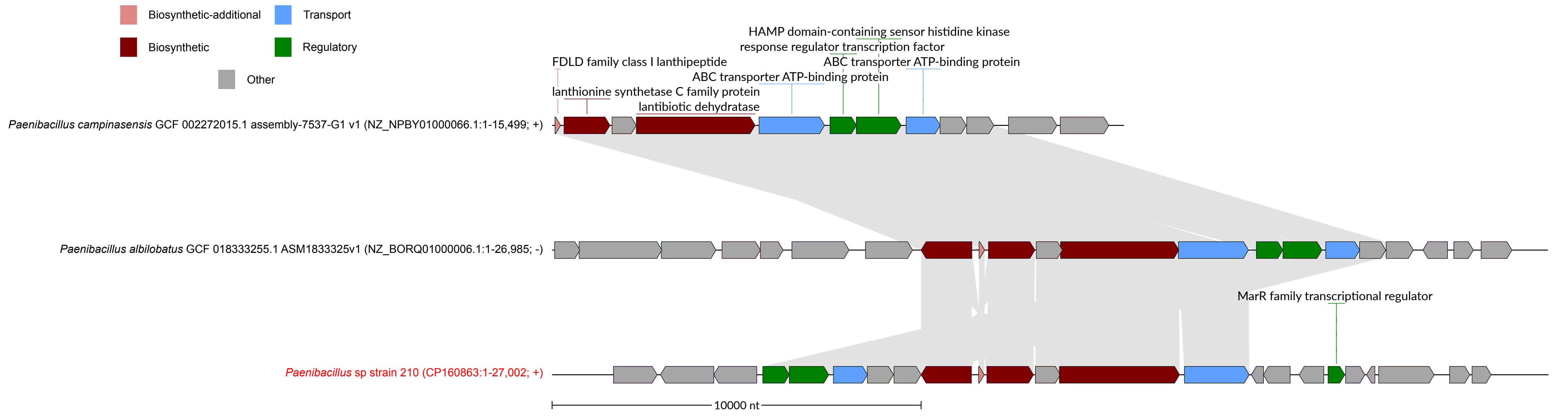

Biosynthetic-additional
Transport
Biosynthetic
Regulatory
Other
HAMP domain-containing sensor histidine kinase
response regulator transcription factor
FDLD family class I lanthipeptide
ABC transporter ATP-binding protein
ABC transporter ATP-binding protein
lanthionine synthetase C family protein
lantibiotic dehydratase
Paenibacillus campinasensis GCF 002272015.1 assembly-7537-G1 v1 (NZ_NPBY01000066.1:1-15,499; +)
Paenibacillus albilobatus GCF 018333255.1 ASM1833325v1 (NZ_BORQ01000006.1:1-26,985; -)
MarR family transcriptional regulator
Paenibacillus sp strain 210 (CP160863:1-27,002; +)
10000 nt
