# Supplementary material for: Genome‐Driven Analysis Reveals the Biotechnological Potential of a Novel Paenibacillus sp. Isolated From Crude Oil
Source: Microbiologyopen. 2025 Nov 24;14(6):e70159. doi: 10.1002/mbo3.70159 (PMC12643537; doi:10.1002/mbo3.70159)
Supplement: Supplementary file 18 — supmat. [file MBO3-14-e70159-s002.docx]

**APPENDIX**

**Figure A1:** Phylogenomic tree assembled using single-copy genes shared between *Paenibacillus* reference genomes. In green highlight, the clade closest to *Paenibacillus* sp. used in comparative analyses.

**Figure A2:** Phylogenomic relationships within the clade containing Paenibacillus sp. strain 210, reconstructed with additional genomes. The analysis confirms its evolutionary placement.

**Figure A3:** Heatmap generated by ClusterANImap, showing the ANI index between genomes of *Paenibacillus* sp. and all reference genomes. Colors range from red (high similarity) to white (low similarity), with gray areas indicating the absence of a relationship. The dendrogram highlights phylogenetic clusters based on genomic similarity.

**Figure A4:** Synteny and collinearity relationships among *Paenibacillus* genomes, shared with *Paenibacillus* sp. strain 210 in regions associated with fusaricidin B biosynthesis. (paeninodin, tridecaptin, paenilan, paenicidin).

**Figure A5:** Synteny and collinearity relationships among *Paenibacillus* genomes, shared with *Paenibacillus* sp. strain 210 in regions associated with paeninodin biosynthesis.

**Figure A6:** Synteny and collinearity relationships among *Paenibacillus* genomes, shared with *Paenibacillus* sp. strain 210 in regions associated with tridecaptin biosynthesis.

**Figure A7:** Synteny and collinearity relationships among *Paenibacillus* genomes, shared with *Paenibacillus* sp. strain 210 in regions associated with paenilan biosynthesis.

**Figure A8:** Synteny and collinearity relationships among *Paenibacillus* genomes, shared with *Paenibacillus* sp. strain 210 in regions associated with paenicidin biosynthesis.

**Figure A9:** Ramachandran plot for the modeled enzymes involved in polysaccharide degradation pathways in strain 210, generated using SAVES 6.1. The plot illustrates the distribution of phi (ϕ) and psi (ψ) dihedral angles, highlighting the favored, allowed, and disallowed regions.

**Table A1:** Summary of reference genomes retrieved from the NCBI RefSeq database.

**Table A2:** Distribution of antibiotic resistance genes detected in genome analysis. Genomes that did not yield results for this analysis were not included in the table.

**Table A3:** Distribution of prophage regions detected in genome analysis. Genomes that did not yield results for this analysis were not included in the table.

**Table A4:** Results for regions linked to secondary metabolite production predicted by antiSMASH and BAGEL4.

**Table A5:** Distribution of CAZy terms detected in genome analysis.

**Table A6:** Metabolic pathway genes labeled and reconstructed by genomic profiling of strain 210.

**Table A7:** Labeled proteins and reference proteins used for comparisons in their primary and tertiary structure, by the predicted models.

**Table A8:** Distribution of genes related to plant growth promotion mechanisms in strain 210 and in the genomes of the closest species according to our phylogenomic results.
