# Supplementary material for: Genome‐Driven Analysis Reveals the Biotechnological Potential of a Novel Paenibacillus sp. Isolated From Crude Oil
Source: Microbiologyopen. 2025 Nov 24;14(6):e70159. doi: 10.1002/mbo3.70159 (PMC12643537; doi:10.1002/mbo3.70159)

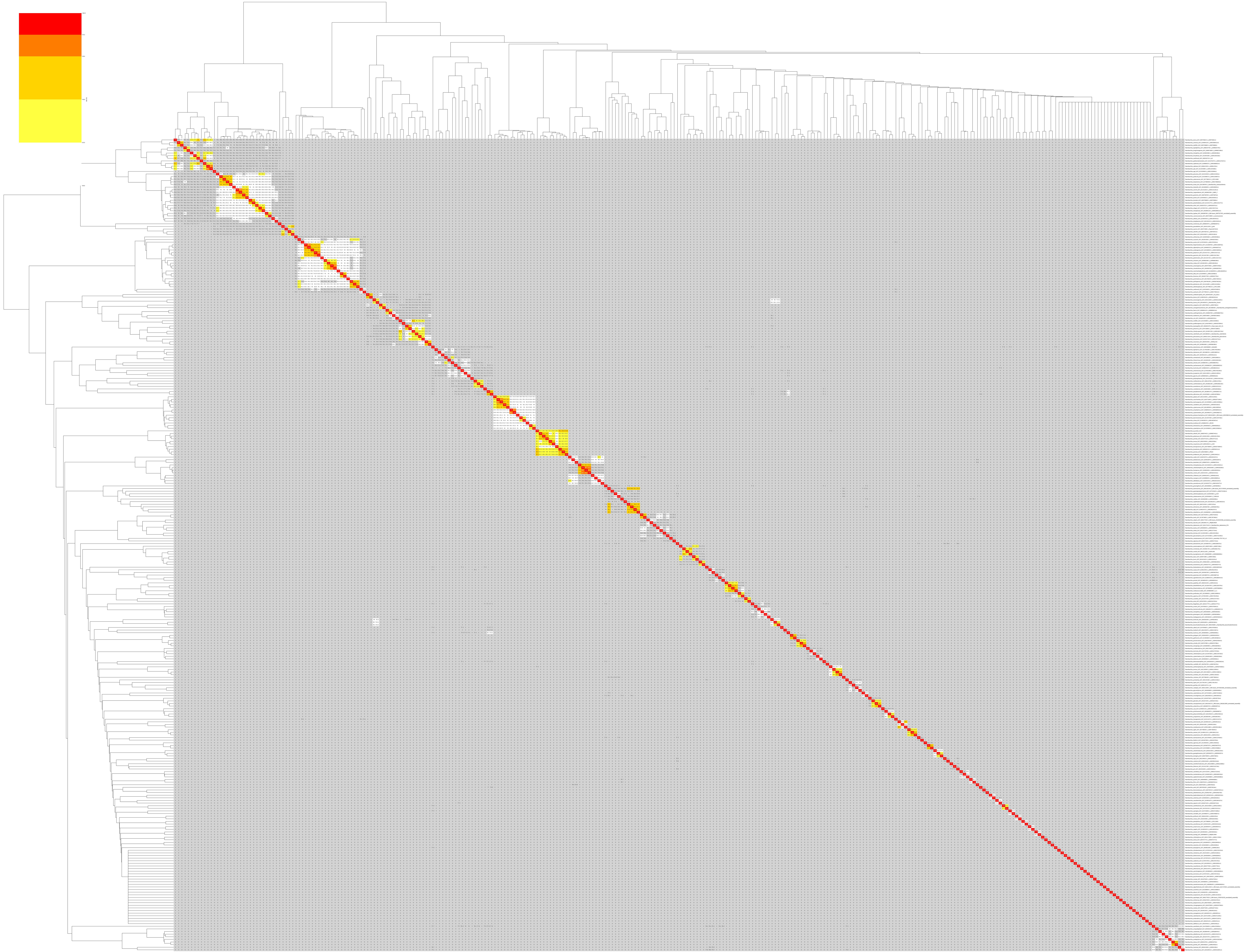

Supplement: Supplementary file 3 — Figure A3: Heatmap generated by ClusterANImap, showing the ANI index between genomes of Paenibacillus sp. and all reference genomes. Colors range from red (high similarity) to white (low similarity), with gray areas indicating the absence of a relationship. The dendrogram highlights phylogenetic clusters based on genomic similarity. [file MBO3-14-e70159-s012.pdf]
